# Systematic proteome-wide Mendelian randomization using the human plasma proteome to identify therapeutic targets for lung adenocarcinoma

**DOI:** 10.1186/s12967-024-04919-z

**Published:** 2024-04-04

**Authors:** Long Zhang, Yajun Xiong, Jie Zhang, Yuying Feng, Aiguo Xu

**Affiliations:** Department of Respiratory and Critical Care Medicine, The First Affiliated Hospital of Zhengzhou University, Zhengzhou, Henan China

**Keywords:** Lung adenocarcinoma, Mendelian randomization, Plasma proteome, Drug target

## Abstract

**Background:**

Lung adenocarcinoma (LUAD) is the predominant histological subtype of lung cancer and the leading cause of cancer-related mortality. Identifying effective drug targets is crucial for advancing LUAD treatment strategies.

**Methods:**

This study employed proteome-wide Mendelian randomization (MR) and colocalization analyses. We collected data on 1394 plasma proteins from a protein quantitative trait loci (pQTL) study involving 4907 individuals. Genetic associations with LUAD were derived from the Transdisciplinary Research in Cancer of the Lung (TRICL) study, including 11,245 cases and 54,619 controls. We integrated pQTL and LUAD genome-wide association studies (GWASs) data to identify candidate proteins. MR utilizes single nucleotide polymorphisms (SNPs) as genetic instruments to estimate the causal effect of exposure on outcome, while Bayesian colocalization analysis determines the probability of shared causal genetic variants between traits. Our study applied these methods to assess causality between plasma proteins and LUAD. Furthermore, we employed a two-step MR to quantify the proportion of risk factors mediated by proteins on LUAD. Finally, protein–protein interaction (PPI) analysis elucidated potential links between proteins and current LUAD medications.

**Results:**

We identified nine plasma proteins significantly associated with LUAD. Increased levels of ALAD, FLT1, ICAM5, and VWC2 exhibited protective effects, with odds ratios of 0.79 (95% CI 0.72–0.87), 0.39 (95% CI 0.28–0.55), 0.91 (95% CI 0.72–0.87), and 0.85 (95% CI 0.79–0.92), respectively. Conversely, MDGA2 (OR, 1.13; 95% CI 1.08–1.19), NTM (OR, 1.12; 95% CI 1.09–1.16), PMM2 (OR, 1.35; 95% CI 1.18–1.53), RNASET2 (OR, 1.15; 95% CI 1.08–1.21), and TFPI (OR, 4.58; 95% CI 3.02–6.94) increased LUAD risk. Notably, none of the nine proteins showed evidence of reverse causality. Bayesian colocalization indicated that RNASET2, TFPI, and VWC2 shared the same variant with LUAD. Furthermore, NTM and FLT1 demonstrated interactions with targets of current LUAD medications. Additionally, FLT1 and TFPI are currently under evaluation as therapeutic targets, while NTM, RNASET2, and VWC2 are potentially druggable. These findings shed light on LUAD pathogenesis, highlighting the tumor-promoting effects of RNASET2, TFPI, and NTM, along with the protective effects of VWC2 and FLT1, providing a significant biological foundation for future LUAD therapeutic targets.

**Conclusions:**

Our proteome-wide MR analysis highlighted RNASET2, TFPI, VWC2, NTM, and FLT1 as potential drug targets for further clinical investigation in LUAD. However, the specific mechanisms by which these proteins influence LUAD remain elusive. Targeting these proteins in drug development holds the potential for successful clinical trials, providing a pathway to prioritize and reduce costs in LUAD therapeutics.

**Supplementary Information:**

The online version contains supplementary material available at 10.1186/s12967-024-04919-z.

## Introduction

Lung cancer is the leading cause of cancer-related deaths, and lung adenocarcinoma (LUAD) is the most common histological subtype, accounting for about 40% of cases [1, 2]. Apart from smoking, other contributors to lung cancer include genetic susceptibility, occupational exposure, air pollution, and chronic lung diseases [3]. Genetic factors are estimated to contribute between 8 and 21% to the heritability of lung cancer [4]. Despite numerous studies shedding light on various aspects of lung cancer pathogenesis [5–7], the identification of new genetic variants associated with the disease remains challenging due to small effect size and the confounding influence of cigarette smoking. To date, only a limited number of lung cancer-specific genes have been discovered [8]. Understanding the genomic architecture of lung cancer is essential for comprehending its pathogenesis and devising personalized targeted therapies. Given that a significant proportion of LUAD patients lack access to targeted therapies, and acquired resistance is common. The novel nanocarrier combined with targeted therapy promotes the development of multimodal targeting for lung cancer [9]. Based on organ-on-a-chip technology, the development of high-throughput drug screening and personalized precision medicine also provides more possibilities for lung cancer treatment [10]. However, the 5-year survival rate for LUAD patients remains at about 23% [11]. Therefore, it is imperative to identify new therapeutic targets for LUAD.

Human proteins play a key role in the development of human diseases and represent the primary targets for approved drugs. A new generation of proteomics technologies has facilitated the identification of abnormal protein expressions, allowing for further exploration of potential biomarkers and therapeutic targets for cancer. Identifying potential proteins associated with LUAD contributes to our understanding of the disease’s underlying mechanisms [12]. In recent years, genome-wide association studies (GWASs) have identified numerous genetic polymorphisms for lung cancer [4, 13]. The goal of GWAS is to identify genetic variations associated with diseases, providing valuable insights into the genetic basis of diseases. GWAS of plasma proteins have revealed genetic variants linked to these proteins, known as “protein quantitative trait loci (pQTLs)” [14]. With the advancement of GWASs in investigating the human plasma proteome, an optimization framework integrating genomic and proteomic databases has emerged for biomarker discovery [15]. Although GWAS have identified many risk loci associated with lung cancer, the genetic underpinnings of the disease remain incompletely understood.

**Fig. 1:**
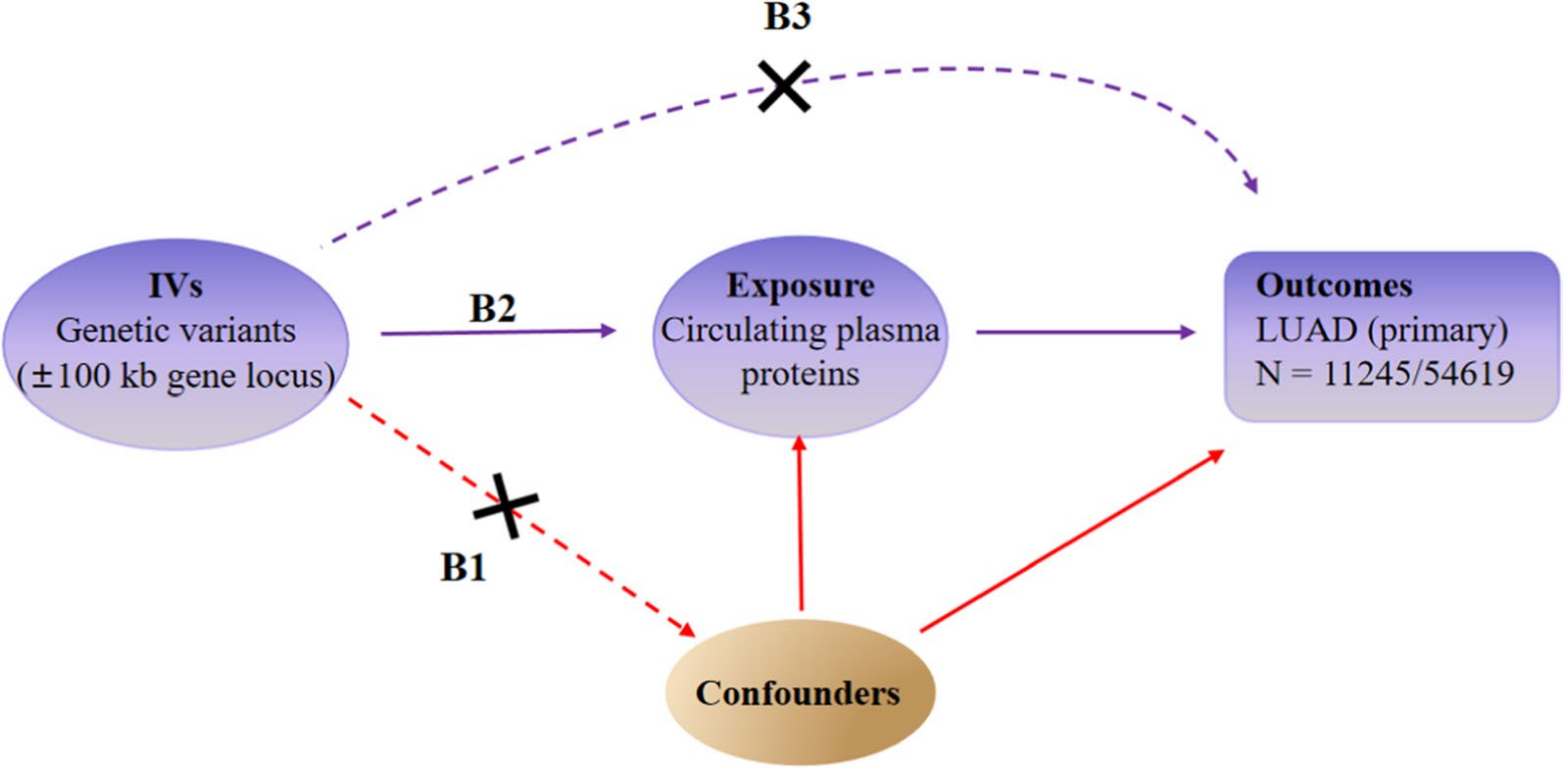
Research overview and design of Mendelian randomization analysis. It should satisfy the following criteria: (1) the IVs are not related to the condounders (**B1**); (2) the IVs are related to the exposure factors (**B2**); (3) the IVs are not directly related to the outcomes (**B3**). IVs: instrumental variables; LUAD: lung adenocarcinama

Due to limitations in traditional study designs, observational stuides cannot fully eliminate the potential for reverse causality and condounding factors, leading to biased associations and conclusions [16]. Mendelian randomization (MR) is a popular approach for causal inferences, utilizing genetic variants as instrumental variables (IVs) that mimic a randomized controlled trial (RCT). MR leverages the random assortment of genetic variants during meiosis, reducing the likelihood of bias from reverse causation or residual confounding [17]. In addition, MR analysis has to fulfill three assumptions (relevance, independence, and exclusion restriction), as detailed in Fig. 1. The exclusion restriction assumption often refers to as the “no pleiotropy assumption”, may be violated in various ways, such as timing effects, interactions, reverse causation, and linkage disquilibrium (LD) [18]. Addressing the horizontal pleiotropy involves selecting IVs with high genetic correlation, employing different analytical methods for result consistency, and utilizing a Bayesian model averaging approach [19]. These strategies are crucial for validating MR findings.

Using MR to analyze GWAS summary data of pQTLs and LUAD provides an oppportunity to identify biological mechanisms related to LUAD and develop novel strategies for prevention. Therefore, our aim is to identify potential plasma proteins associated with LUAD.

**Fig. 2:**
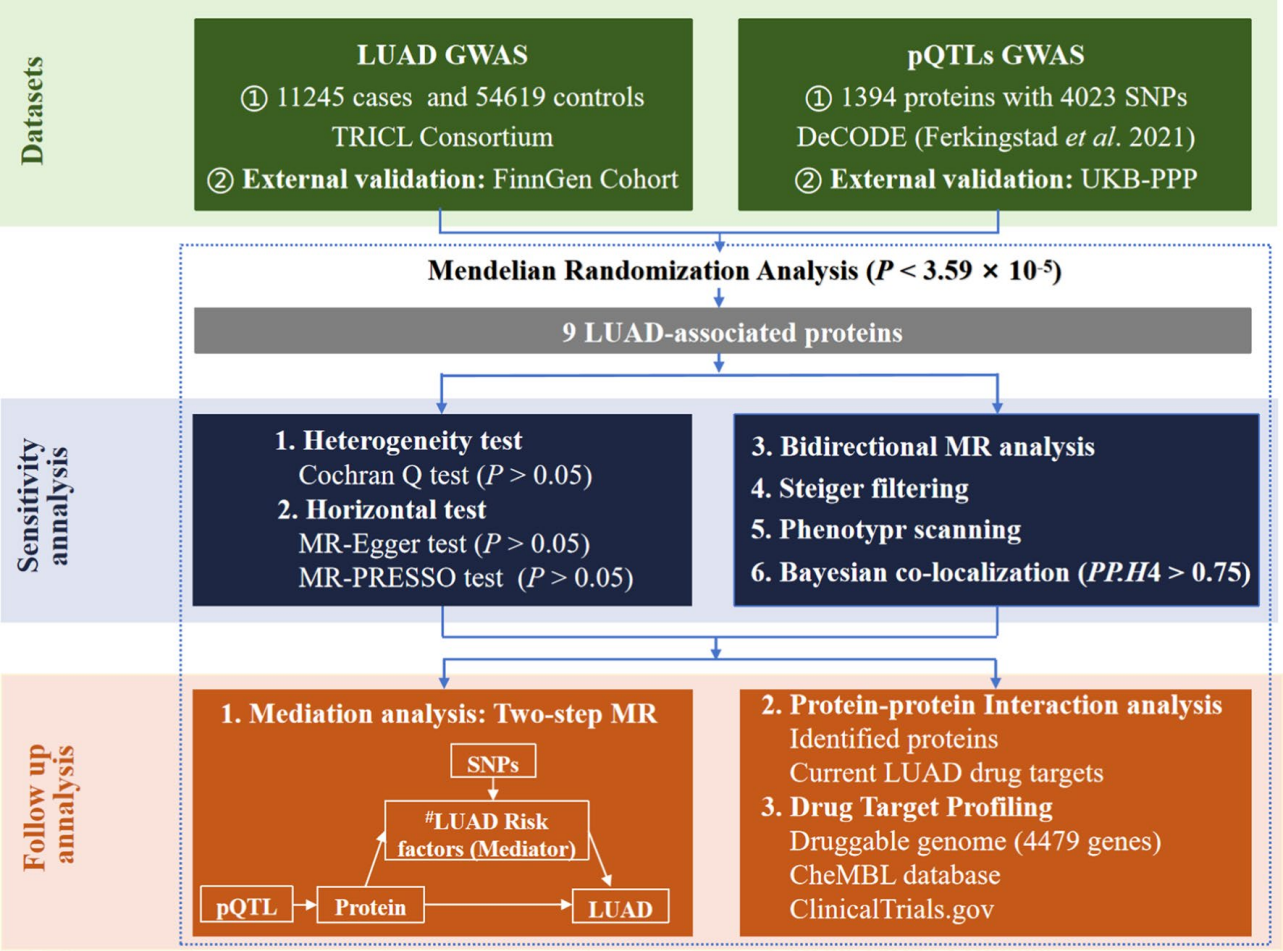
Study design. First, using MR to identify potential causal proteins for LUAD by utilizing plasma pQTL data from Ferkingstad’s study and GWAS data from the TRICL Consortium. Otherwise, we replicated the primary analysis in dependent cohorts, using plasma pQTL data from Sun’s study and LUAD GWAS data from FinnGen Cohort. Second, sensitivity analyses were used to validate our primary findings, including Cochran Q test, MR-Egger test, and MR-PRESSO test. Bidirectional MR analysis and Steiger filtering were used to ensure the directionality of causality. Phenotype scanning and Bayesian co-localization were employed to detect potential horizontal pleiotropy. Third, we conducted mediation analysis using a two-step MR for proteins displaying potential causality with both LUAD and risk factors. Fourth, we mapped the interaction network among the identified proteins and their associations with the targets of current LUAD drugs. Lastly, we searched for an updated list of druggable genes, the ChEMBL database and a clinical trials registry website to evaluate the druggability of the identified proteins. ^#^LUAD Risk factors: age of smoking initiation, number of cigarettes smoked daily, pack years of smoking, maternal smoking around birth, any parental history of lung cancer, average weekly beer intake, leisure activities, and chronic obstructive pulmonary disease (COPD). LUAD: lung adenocarcinoma; GWAS: genome-wide association study; TRICL: Transdisciplinary Research in Cancer of the Lung; pQTLs: protein quantitative trait locus; LD: linkage disequilibrium; SNPs: single nucleotide polymorphisms; FDR: false discovery rate; MR: Mendelian Randomization; MR-PRESSO: MR Pleiotropy Residual Sum and Outlier; PP.H4: posterior probability of *H*4

In this study, we obtained genetic instrumental variables for plasma pQTL data from Ferkingstad’s study [14] and GWAS data for LUAD from the Integrative Epidemiology Unit (IEU) Open GWAS Project [20–24]. The study design is depicted in Fig. 2. We initially performed MR to estimate the causal effect of plasma proteins on LUAD, identifying nine LUAD-associated proteins. We replicated our analysis using plasma pQTL data from the study by Sun et al. [25] and GWAS data from the FinnGen cohort [26] for external validation. To ensure result reliability, we performed sensitivity analyses, Bayesian colocalization analysis, reverse causality detection, and phenotype scanning. Next, we constructed a protein–protein interaction network linking the identified proteins with the current targets of LUAD medications. Finally, we assessed the causal effect of plasma proteins on LUAD risk factors and quantified the proportion of the protein’s effect on LUAD mediated by these risk factors. The identified proteins offer promising targets for novel interventions through their specific interactions and regulatory roles in key cellular pathways. Discovering the complex mechanisms by which these proteins influence LUAD pathogenesis could provide valuable insights for the development of innovative therapeutic strategies.

## Materials and methods

### Data sources

We obtained pQTL data from a study conducted by Ferkingstad et al.[14]. These pQTL should satisfy the following criteria: (a) demonstrating genome-wide significant association (*P* < 5 × 10^–8^); (b) showing independent association (linkage disquilibrium (LD) clumping r^2^ < 0.001); and (c) being cis-pQTL. Based on these criteria, we identified 4144 SNPs associated with 1394 proteins. Additional file 2: Table S1 provides details regarding these 1394 proteins.

For the primary outcome, we found summary genetic association data for LUAD from the IEU Open GWAS Project (https://gwas.mrcieu.ac.uk/). Relevant datasets from 2014 to 2023 were explored using search terms such as “lung cancer” and “lung adenocarcinoma”. Inclusion and exclusion criteria were applied to select studies aligning with our research objectives. Finally, we obtained a dataset consisting of individuals of European ancestry, including 11,245 LUAD cases and 54,619 controls [20–24]. Table 1 provides details about the data sources used in this study.

**Table 1:** Data sources for the Mendelian randomization analysis in the study

Phenotype	Sample size	Ancestry	Sources
Plasma protein			
deCODE (primary)	4907	Icelander	Ferkingstad, et al. [14]
UKB-PPP (validation)	33,469	European	Sun et al. [25]
Outcomes			
LUAD (primary)	11,245/54619	European	TRICL [20–24]
LUAD (validation)	1553/287137	European	FinnGen Cohort [26]
Risk factors			
Age of smoking initiation	341,427	European	Liu, et al. [27]
Number of cigarettes smoked daily	108,946	European	UK Biobank
Pack years of smoking	142,387	European	UK Biobank
Maternal smoking around birth	121,634/276098	European	UK Biobank
Any parental history of lung cancer	407,521	European	Mbatchou J [28]
Average weekly beer intake	327,634	European	UK Biobank
Leisure activities	67,877/393492	European	UK Biobank
COPD	13,530/454945	European	Sakaue et al. [29]

Samplesize shown as a total number for quantitative traits and cases/controls for binary traits

TRICL: Transdisciplinary Research in Cancer of the Lung; PLCO: Prostate, Lung, Colorectal, and Ovarian Cancer Screening Trial. LUAD: lung adenocarcinoma; COPD: chronic obstructive pulmonary disease

In addition, we used plasma pQTL data from the study by Sun et al. [25], which included 2923 plasma proteins with 33,469 participants, and LUAD GWAS data obtained from the FinnGen study (ncase = 1553, ncontrol = 287,137, R9 release) [26] for external validation.

### Statistics analysis

#### Mendelian randomization analysis

We employed the R package “TwoSampleMR” (version 0.5.7) for MR analysis to estimate the associations between genetically predicted protein levels and LUAD. The Wald ratio method was applied for proteins with only one pQTL, while for those with two or more pQTLs, we used the inverse-variance-weighted (IVW), MR Egger, weighted median, simple mode, and weighted mode to estimate the causality, of which the IVW is the most important method [30]. Odds ratios (OR) for increased risk of LUAD were expressed as per standard deviation (SD) increase in plasma protein levels. The study frame chart of MR analysis is presented in Additional file 1: Fig. S1. To minimize the probability of false positives, we implemented Bonferroni correction for multiple testing, setting a significant threshold at *P*value of 0.05/1394 (*P* < 3.59 × 10^–5^).

#### Sensitivity analysis

We conducted a series of sensitivity analyses to validate the robustness of our findings. First, we used the Cochran Q test to estimate the heterogeneity of genetic variants, and it indicated no heterogeneity when *P* > 0.05[31]. Second, MR-Egger’s intercept was utilized to evaluate horizontal pleiotropy, with *P* > 0.05 showing no horizontal pleiotropy [32]. Third, we employed MR Pleiotropy Residual Sum and Outlier (MR-PRESSO) to identify influential outlier IVs due to pleiotropy, where *P* > 0.05 for the Global test indicated the presence of horizontal pleiotropic outliers [33].

When genetic variants influence the exposure through the outcome, it can lead to incorrect inferences about causality, particularly in complex biological processes or mutually influencing factors. To enhance the robustness of the causal relationship, we performed bidirectional MR analysis, using LUAD as the exposure and pQTL as the outcome. This approach helps detect potential reverse causality, avoid confounding factors and ensure the integrity of our genetic association study [34]. Moreover, we conducted Steiger filtering to confirm the direction of associations between identified proteins and LUAD. The Steiger filtering assumes that a valid IV should explain more variation in the exposure than in the outcome. If an IV satisfies this criterion, its direction is “TRUE”; otherwise, it is “FALSE”. After removing SNPs with “FALSE” direction, we repeated all MR analyses using the IVW method [35]. *P* < 0.05 was considered statistically significant.

Furthermore, we conducted phenotype scanning, reviewing prior GWAS to unveil associations between identified pQTLs and other characteristics [33]. An SNP was considered pleiotropic if it was associated with recognized risk factors for LUAD, including but not limited to smoking, exposure to air pollutants, occupational carcinogens, alcohol consumption, leisure activities, and chronic pulmonary conditions.

#### Bayesian co-localization analysis

We conducted colocalization analysis using the R package “coloc” to determine if the associations between the identified proteins and LUAD resulted from linkage disequilibrium [36]. Bayesian co-localization assigns posterior probabilities to five hypotheses: PP.H_0_, no association with either plasma protein or LUAD; PP.H_1_, association with plasma protein only; PP.H_2_: association with LUAD only; PP.H_3_, association with both plasma protein and LUAD, but with distinct causal variants; PP.H_4_, association with both plasma protein and LUAD, with the same causal variant. Two signals were considered to have strong evidence of colocalization if the posterior probability for a shared causal variant (PP.H4) ≥ 0.75, and medium colocalization indication was defined as 0.5 < PP.H4 < 0.75. Proteins with high support evidence of colocalization (PP.H4 ≥ 0.75) were classified as tier 1 targets, proteins with medium support evidence of colocalization (0.5 < PH4 < 0.75) were classified as tier 2 targets, and the remaining proteins were classified as tier 3 targets [37].

#### Mediation analysis

We identified LUAD risk factors through a comprehensive literature review [3, 38]. Tobacco smoking is recognized as the major cause of LUAD.In addition to smoking, other factors such as genetic susceptibility, chronic obstructive pulmonary disease (COPD), occupational exposures, air pollution, alcohol consumption, and physical activity may independently or collaboratively contribute to the descriptive epidemiology of LUAD. We excluded risk factors without GWAS data, like air pollution. Subsequent MR analysis revealed no causal relationship between certain factors, such as occupational exposures, leading us to focus our further analysis on the retained factors. These included smoking variables (age of smoking initiation, number of cigarettes smoked daily, and pack years of smoking), maternal smoking around birth, any parental history of lung cancer, average weekly beer intake, leisure activities, and COPD. Detailed information regarding the GWAS summary data of LUAD risk factors is provided in Table 1.

We conducted mediation analyses to quantify the effect of identified proteins on LUAD via risk factors using a two-step MR method. The total effect of exposure on outcome can be broken down into direct effects and indirect effects [39]. In our study, the total effects of identified proteins on LUAD comprised: (1) the direct effects of identified proteins on LUAD, calculated by primary MR; (2) the indirect effects mediated through the mediator, estimated using the Product method. Standard error (SE) and confidence interval (CI) were determined using the delta method [39].

### Evaluation of druggability

To explore interations between potential therapeutic targets and the underlying mechanisms of LUAD, we performed PPI analysis involving the identified plasma proteins and previously recognized drug therapeutic targets. The PPI analysis was carried out using the STRING database (https://string-db.org) [40] and Cytoscape software, with a minimum required interaction score of 0.4 [41].

Furthermore, we evaluated the druggability of the candidate target proteins by referencing Finan’s study on 4479 druggable genes [42], the ChEMBL database (https://www.ebi.ac.uk/chembl) [43], and ClinicalTrials (https://www.ClinicalTrials.gov). These 4479 druggable genes were categorized into three tiers, tier 1 included 1427 efficacy targets of approved drugs and clinical-phase drug candidates, tier 2 encompassed 682 targets closely linked to approved drug targets or drug-like compounds, and tier 3 comprised 2370 targets with more distant similarities to approved drug targets. We obtained information about compound names, molecule types, and action types of the targeted proteins from ChEMBL. Additionally, we searched ClinicalTrials to gather data on the clinical phases of the target proteins under development.

### Ethical statement

No ethical approval was required for the present study, for all data sources were based on publicly available summary-level data. All these studies were approved by the relevant institutional review committees.

## Results

### Screening the proteome for lung adenocarcinoma causal proteins

**Fig. 3:**
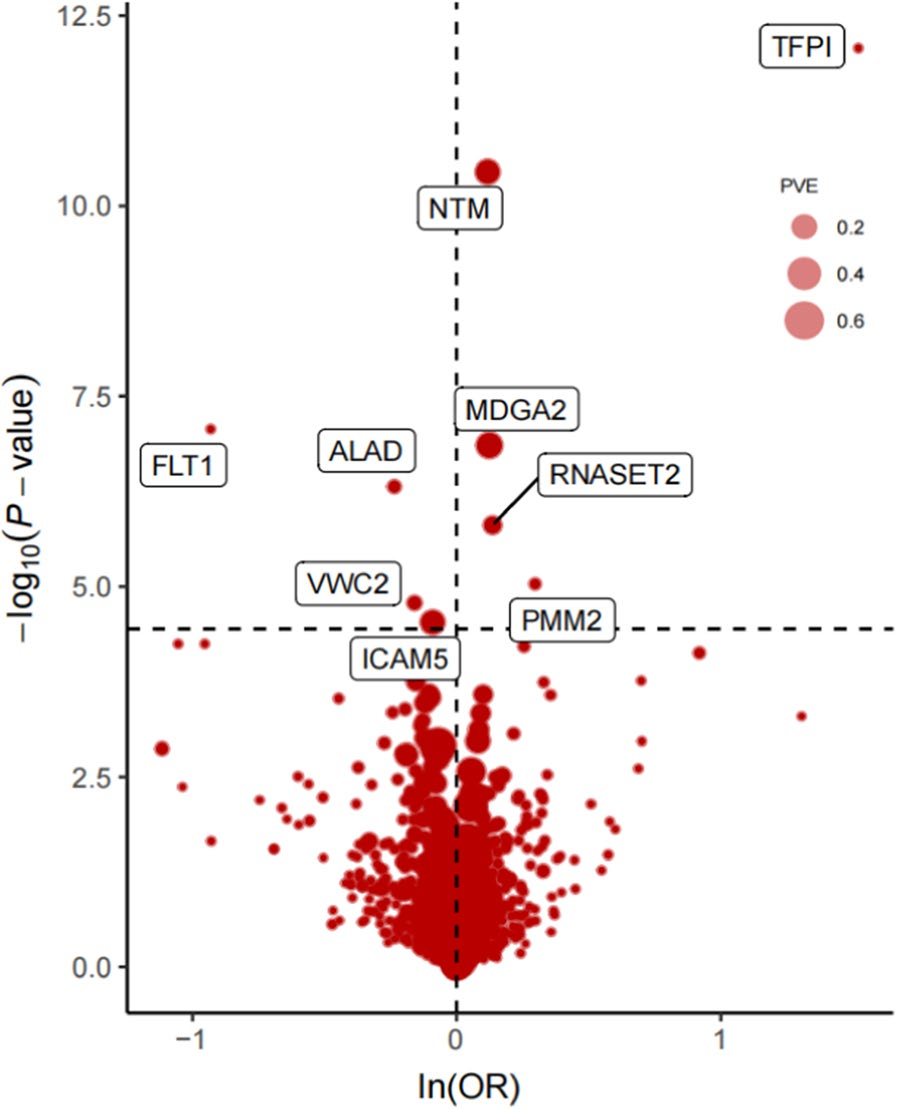
Volcano plot of the MR results for 1394 plasma proteins on LUAD. OR for increased risk of LUAD were expressed as per SD increase in plasma protein levels. Dashed horizontal black line corresponded to *P* = 3.59 × 10^−5^ (0.05/1394). ln = natural logarithm; PVE = proportion of variance explained. OR: odds ratio; LUAD: lung adenocarcinoma; SD: standard deviation

**Fig. 4:**
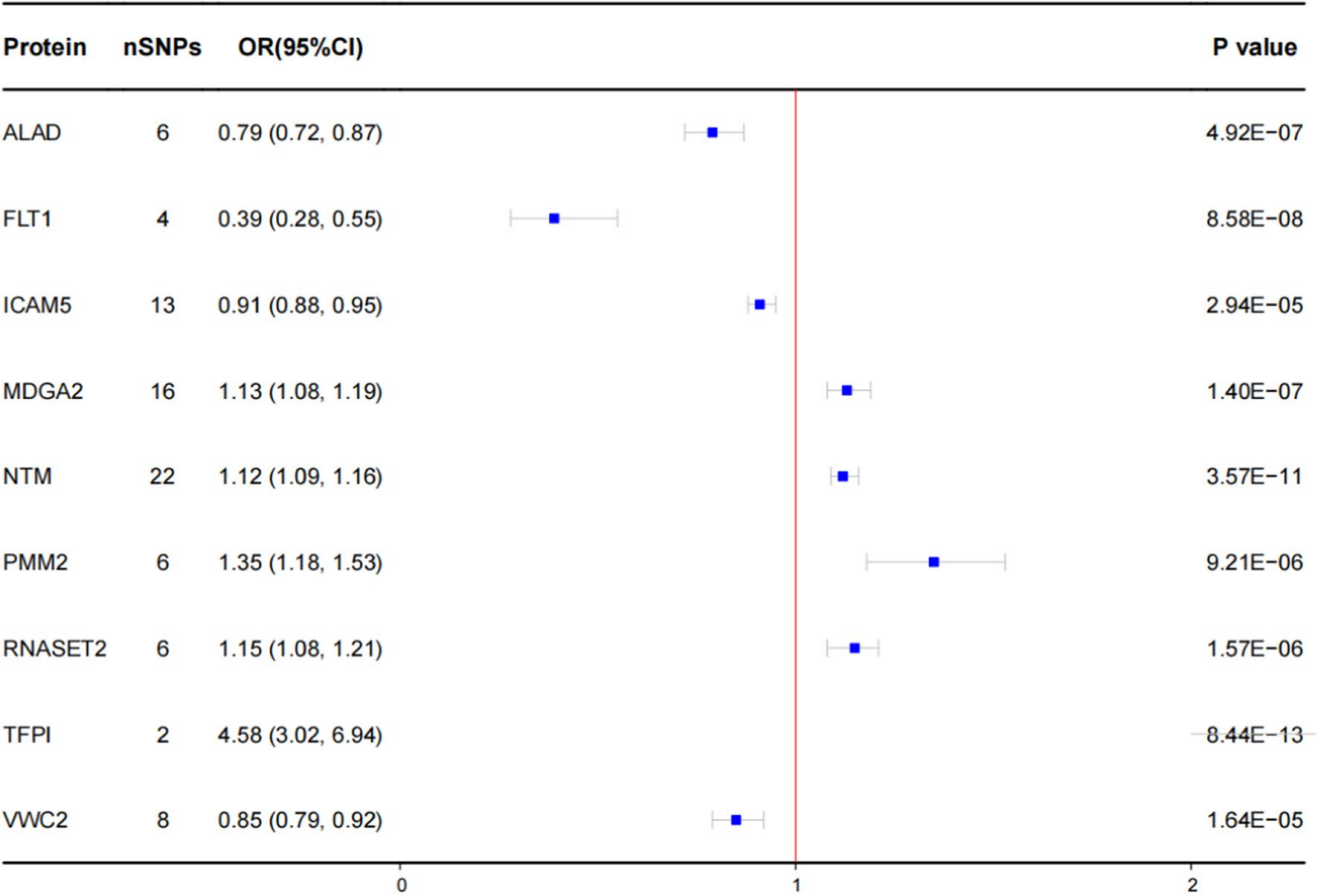
MR analyses for nine potential causal proteins on LUAD. The squares were the causal estimates on the OR scale, and the whiskers represented the 95% CI for these ORs. nSNPs: number of SNPs used for the estimation of the causal effects in this plot. OR for increased risk of LUAD were expressed as per SD increase in plasma protein levels. *P* values were determined from the IVW MR method. OR: odds ratio; CI: confidence interval; LUAD: lung adenocarcinoma; SD: standard deviation; IVW: inverse-variance-weighted; MR: Mendelian randomization

MR analysis identified nine plasma proteins associated with LUAD (Figs. 3, 4 and Additional file 1: Fig. S2). These proteins include aminolevulinate dehydratase (ALAD), Fms-related receptor tyrosine kinase 1 (FLT1), intercellular adhesion molecule 5 (ICAM5), MAM domain-containing glycosylphosphatidylinositol anchor 2 (MDGA2), neurotrimin (NTM), phosphomannomutase 2 (PMM2), ribonuclease T2 (RNASET2), tissue factor pathway inhibitor (TFPI), and von Willebrand factor C domain- containing 2 (VWC2). Specifically, ALAD (OR = 0.79; 95% CI 0.72–0.87; *P* = 4.92 × 10^–7^), FLT1 (OR = 0.39; 95% CI 0.28–0.55; *P* = 8.58 × 10^−8^), ICAM5 (OR = 0.91; 95% CI 0.88–0.95; *P* = 2.94 × 10^−5^), and VWC2 (OR = 0.85; 95% CI 0.79–0.92; *P* = 1.64 × 10^−5^) were associated with a decreased risk of LUAD, while MDGA2 (OR = 1.13; 95% CI 1.08–1.19; *P* = 1.40 × 10^–7^), NTM (OR = 1.12; 95% CI 1.09–1.16; *P* = 3.57 × 10^−11^), PMM2 (OR = 1.35; 95% CI 1.18–1.53; *P* = 9.21 × 10^−6^), RNASET2 (OR = 1.15; 95% CI 1.08–1.21; *P* = 1.57 × 10^−6^), and TFPI (OR = 4.58; 95% CI 3.02–6.94; *P* = 8.44 × 10^−13^) were associated with an increased risk of LUAD (Additional file 2: Table S2, S3).

We validated our primary findings in other datasets, RNASET2 was also identified as being associated with LUAD in the FinnGen cohort. Using a genome-wide significant variant reported by Sun et al. as a genetic instrument, RNASET2 increased the risk of LUAD (OR = 1.20; 95% CI 1.03–1.39; *P* = 0.02), while other proteins showed no association with LUAD. ALAD, MDGA2, and NTM were not found in the UKB-PPP dataset (Additional file 1: Fig. S3).

### Sensitivity analysis for lung adenocarcinoma causal proteins

The results of sensitivity analyses confirmed the robustness of our primary MR analyses. No evidence of heterogeneity was found in the association of the nine proteins in Additional file 2: Table S3, as measured by Cochran Q statistics (*P*_Q-stat_ > 0.05). And there was no horizontal pleiotropy in the IVs, as assessed by MR-Egger intercept (*P*_Egger-Intercept_ > 0.05) or MR-PRESSO global pleiotropy test (*P*_GlobalTest_ > 0.05). Bidirectional MR analysis revealed no evidence of reverse causations (Additional file 1: Fig. S4) and Steiger filtering ensured directionality (Additional file 2: Table S4). The high support of colocalization evidence was observed between 3 proteins (RNASET2, TFPI, and VWC2) and LUAD, which were identified as tier 1. ALAD was identified as tier 2 due to its medium support of colocalization evidence. The remaining proteins with limited evidence of colocalization were ascertained as tier 3 targets (Additional file 2: Table S5 and Additional file 1: Fig. S5). Finally, after phenotype scanning, RNASET2 (rs71573407) was linked to complications of the puerperium, while RNASET2 (rs162298) was associated with Crohn’s disease, hypothyroidism or myxoedema, and treatment with levothyroxine sodium. VWC2 (rs10282410) was associated with sitting height (Additional file 2: Table S6). Of which, the relationship between Crohn’s disease and LUAD has sparked great interest. Bobby et al. [44] reported that Crohn’s disease was associated with a higher risk of LUAD (OR = 1.53, 95% CI 1.23–1.91, *P* < 0.05). After removing rs162298, the causality between RNASET2 and LUAD remained significant (IVW OR = 1.12, 95% CI 1.04–1.20, *P* = 0.003). Regarding other traits, none of the associations could fully elucidate the connection between identified proteins and LUAD. The findings suggested that the causality between identified proteins and LUAD was not violated by potential risk factors.

### Identification of likely causal LUAD risk factors

To understand potential mechanisms between proteins and LUAD, we performed a two-step mediation MR involving conventional LUAD risk factors. Firstly, we evaluated the causal relationship between these risk factors and LUAD. Subsequently, we examined the causal effects of the identified proteins on the risk factors.

**Fig. 5:**
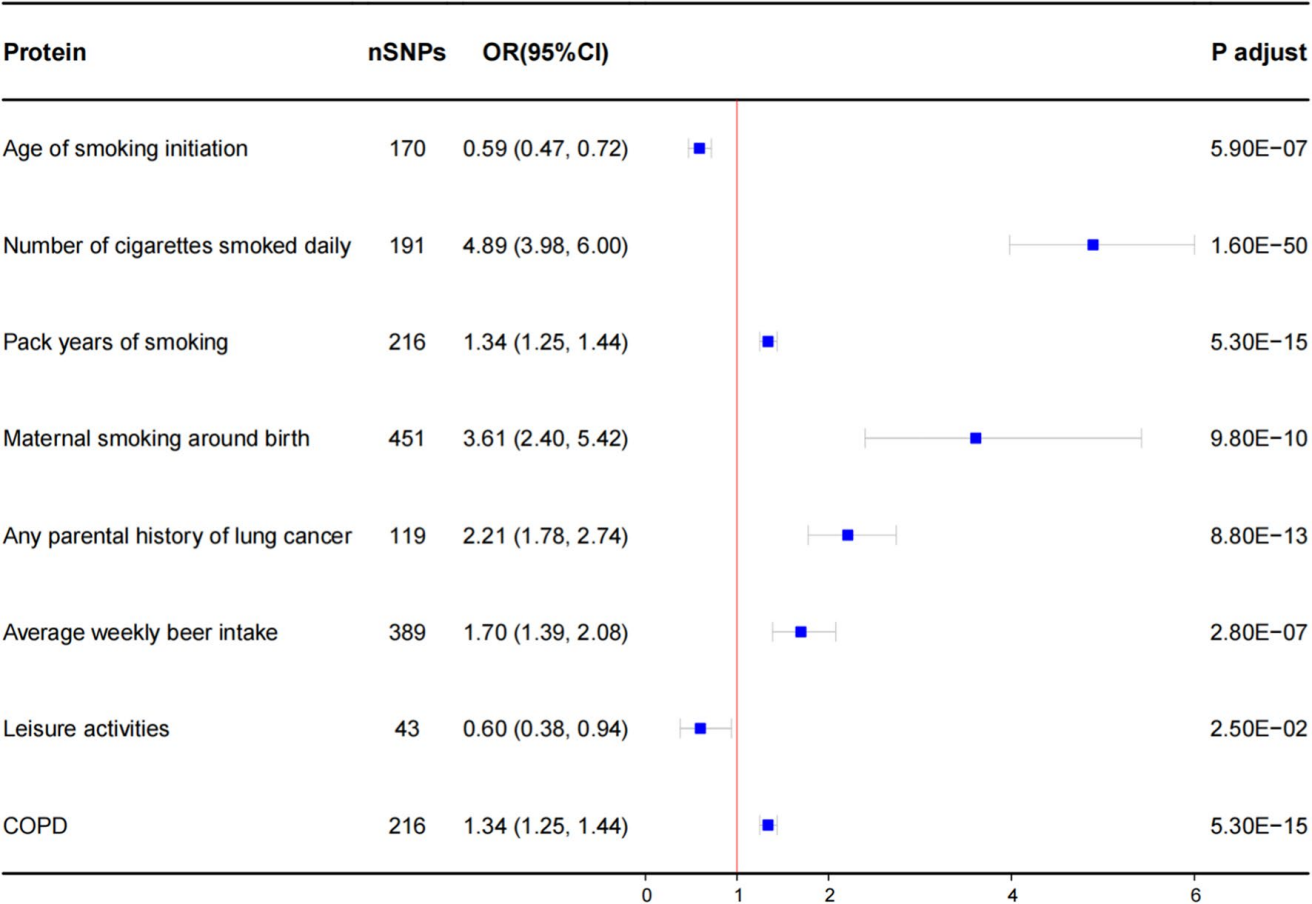
Causal effects of risk factors on LUAD through MR analyses. The squares are the causal estimates on the OR scale, and the whiskers represent the 95% CI for these ORs. nSNPs: number of SNPs used for the estimation of the causal effects in this plot. OR per SD increase in plasma protein levels as LUAD risk increased. *P* values were determined from the IVW MR method. OR: odds ratio; CI: confidence interval; COPD: chronic obstructive pulmonary disease; LUAD: lung adenocarcinoma; MR: Mendelian randomization; SD: standard deviation; IVW: inverse-variance-weighted

As anticipated, smoking, maternal smoking around birth, any parental history of lung cancer, alcohol consumption, and COPD increased the risk of LUAD, while the age of smoking initiation and engagement in lesisure activities were linked to a reduced risk of LUAD (Fig. 5). In particular, the number of cigarettes smoked daily (OR = 4.89; 95% CI 3.98–6.00; *P* = 1.60 × 10^−50^) and pack years of smoking (OR = 1.34; 95% CI 1.25–1.44; *P* = 5.30 × 10^−15^) were associated with an increased risk of LUAD. Maternal smoking around birth (OR = 3.61; 95% CI 2.40–5.42; *P* = 9.80 × 10^−10^) was also linked to a higher LUAD risk. Notably, the average weekly beer intake (OR = 1.70; 95% CI 1.39–2.08; *P* = 2.80 × 10^−07^) exhibited a strong association with LUAD. Genetically determined higher COPD risk (OR = 1.34; 95% CI 1.25–1.44; *P* = 5.30 × 10^−15^) was also correlated with an increased LUAD risk. On the other hand, an increasing age of smoking initiation was associated with a reduced risk of LUAD (OR = 0.59; 95% CI 0.47–0.72; *P* = 5.90 × 10^−07^), and engagement in leisure activities (OR = 0.60; 95% CI 0.38–0.94; *P* = 2.50 × 10^−02^) decreased the risk of LUAD (Additional file 2: Table S7).

### Identification of LUAD risk factors associated proteins and mediation analysis

We performed MR on three plasma proteins (RNASET2, TFPI and VWC2) in relation to LUAD risk factors (Additional file 2: Table S8). Our results revealed that genetically elevated TFPI levels were associated with an increased daily number of cigarettes smoked (OR = 1.017; 95% CI 1.003–1.030; *P* = 0.015) and pack years of smoking (OR = 1.020; 95% CI 1.000–1.030; *P* = 0.025). Furthermore, higher genetically determined VWC2 levels were associated with greater engagement in leisure activities (OR = 1.004; 95% CI 1.002–1.006; *P* = 0.000).

To explore the proteins’ indirect impact on LUAD through risk factors, we performed a mediation analysis using effect estimates from a two-step MR and the total effect from the primary MR. The proportion of mediation effect of TFPI via number of cigarettes smoked daily and pack years of smoking was 2.70% and 1.30%, respectively. The indirect effect of VWC2 on LUAD risk through leisure activities accounted for 1.20% (Additional file 2: Table S9).

### Association of potential drug targets with current LUAD medications.

**Fig. 6:**
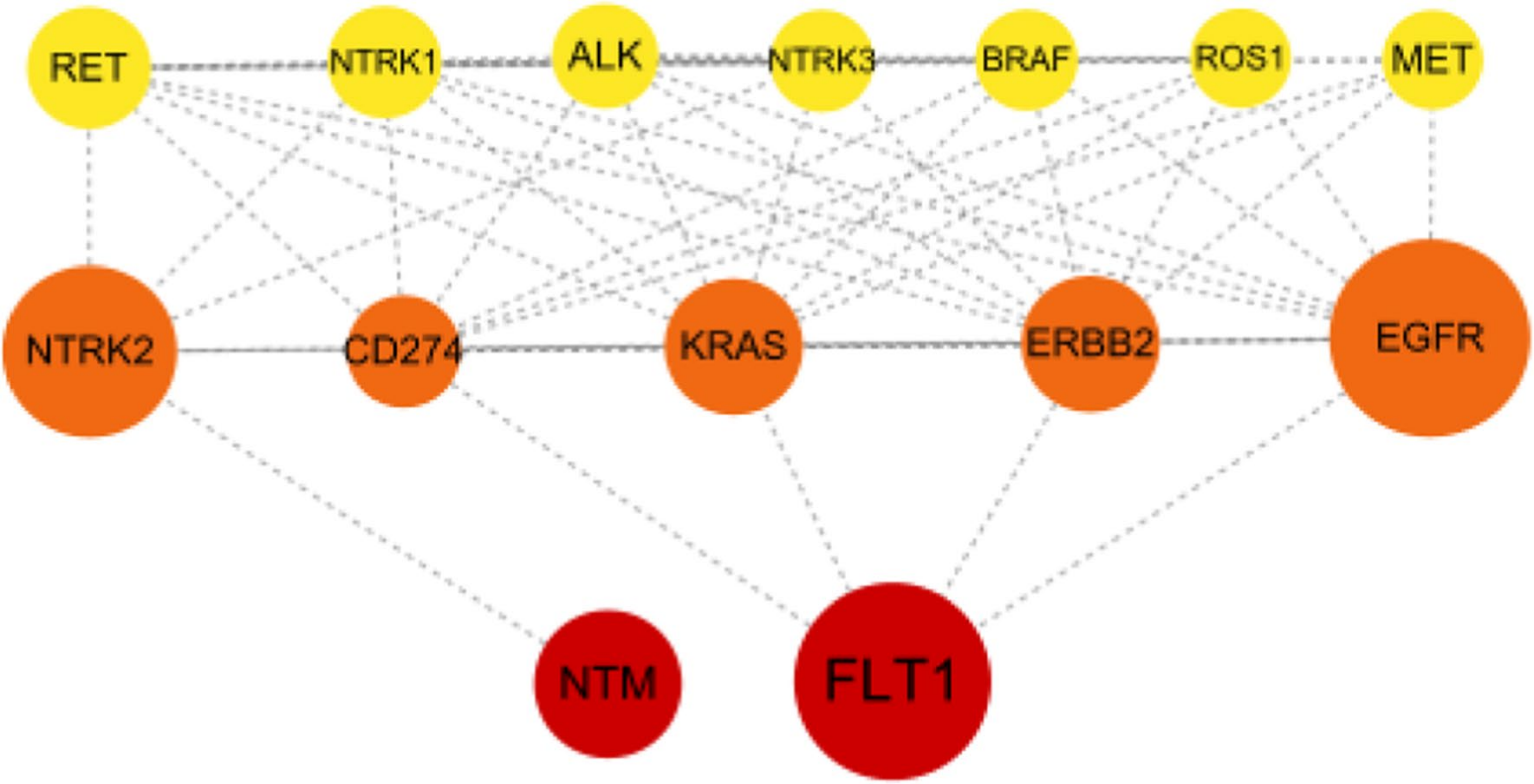
Protein–Protein interaction network among the causal proteins and current medication targets for LUAD. Red circles represented plasma proteins (FLT1 and NTM). Orange solid circles depicted current LUAD medication targets associated with potential proteins, while yellow solid circles represented current LUAD medication targets without such associations. The size of the circle indicated the number of interacting proteins. LUAD: lung adenocarcinoma

**Fig. 7:**
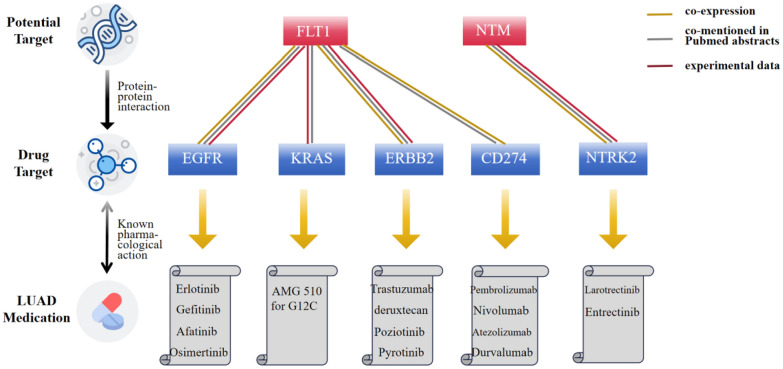
Interaction between identified plasma proteins and current medication targets for LUAD. LUAD: lung adenocarcinoma

The PPI network revealed interactions between two identified proteins (FLT1 and NTM) and the targets of five current LUAD medications (Figs. 6, 7). Specifically, FLT1 was associated with epidermal growth factor receptor (EGFR), the target of Erlotinib, Gefitinib, Afatinib, and Osimertinib. FLT1 was also linked to Kirsten Rat Sarcoma Viral Oncogene Homolog (KRAS), Erb-B2 Receptor Tyrosine Kinase 2 (ERBB2), and cluster of differentiation 274 (CD274), also known as programmed death-ligand 1 (PD-L1). KRAS is the target of AMG 510 for G12C. ERBB2 is the target of Trastuzumab, deruxtecan, Poziotinib, and Pyrotinib. CD274 (PD-L1) is the target of Pembrolizumab, Nivolumab, Atezolizumab and Durvalumab. NTM was associated with neurotrophic tyrosine kinase receptor type 2 (NTRK2), the target of Larotrectinib and Entrectinib (Additional file 1: Fig. S6).

### Druggability and clinical‑phase drug for candidate protein targets

We conducted a comprehensive investigation, utilizing a list of druggable genes [42], the ChEMBL database [43], and the ClinicalTrials website, to assess the druggability and drug development of the nine plasma proteins. We categorized the potential targets into three groups: (1) in development (currently in clinical trials); (2) druggable (listed as druggable targets); and (3) not currently recognized as druggable. Notably, the FLT1-targeted drug FAMITINIB was entering phase I/II trials for various cancers, including NSCLC, breast cancer, colorectal cancer, renal cell cancer, and others. Additionally, the FLT1-targeted drug ICRUCUMAB was in phase II trials for bladder, urethra, ureter, or renal pelvis carcinoma. TFPI-targeted drugs CONCIZUMAB, MARSTACIMAB, ANDEXANET ALFA, and BAY-1093884 were undergoing phase I/II/III trials for hemophilia (Additional file 2: Table S10). Although there are currently no ongoing trials for NTM, RNASET2, and VWC2, they remain potential druggable targets. While ALAD, ICAM5, MDGA2, and PMM2 are not currently listed as potential drug targets.

## Discussion

We used plasma pQTL data to identify potential therapeutic targets for LUAD, revealing nine proteins. A “causality” identified by MR might be influenced by reverse causality, horizontal pleiotropy or confounding factors [45]. Strong evidence of an association between genetically predicted levels of RNASET2, TFPI, and VWC2 and LUAD emerged from colocalization analysis. Consequently, bidirectional MR was conducted and no proteins revealed reverse causality, which was further supported by Steiger filtering. Moreover, we listed two protein-targeted drugs in development (FLT1 and TFPI) and three druggable proteins (NTM, RNASET2, and VWC2).

Our findings indicate that smoking, maternal smoking around birth, parental history of lung cancer, alcohol consumption, leisure activities, and COPD are causally associated with LUAD, highlighting their roles in LUAD pathogenesis and aligning with classical epidemiological studies [3]. A positive family history of lung cancer has been identified as a risk factor in studies reporting a high familial risk for early-onset lung cancer. Exposure to tobacco smoke during pregnancy may significantly increase the incidence and mortality of lung cancer in adulthood [46]. Consuming 30g or more of alcohol per day is linked to an increased risk of lung cancer compared to non-alcohol consumption, with the risk being particularly pronounced in male never smokers [47]. Some studies have also proposed an inverse relationship between physical activity and lung cancer risk [38]. However, the evidence has been limited to current and former smokers in most stuides. Traditional observational stuides are susceptible to confounding and reverse causation. Baumeister et al. [48] found that physical activity had no effect on lung cancer through MR in a large cohort.

TFPI functions as a serine protease inhibitor, affecting prothrombin clearence via the Tissue Factor (TF) pathway [49]. TF, known for its role in promoting tumor angiogenesis and metastasis, can drive cancer progression [50]. The role of TFPI in cancer remains contentious. TFPI was deemed a tumor suppressor initially. TFPI was observed to induce invasive tumor growth upon silencing in breast cancer cells, while its overexpression triggered apoptosis [51]. Mice lacking TFPI exhibited increased metastatic potential [52]. Our findings align with emerging evidence suggesting TFPI might actively contribute to tumor progression. Elevated TFPI expression has been noted in various invasive tumors [51]. Arnason et al. [52] found that TFPI facilitated multiple drug resistance (MDR) in cancer cells. Phenoscanner revealed associations between TFPI SNP(rs116350534) and LUAD [24]. Our study further identified higher TFPI levels among individuals with a smoking history. TFPI has exhibited potential in differentiating LUAD among high-risk smokers [53], with research indicating increased TFPI activity in smokers [54]. Such elevated TFPI activity in smokers could arise from endothelial damage or chronic inflammation in vessel walls, leading to increased TFPI synthesis, release, or binding to the endothelium [55]. Nevertheless, establishing a direct link between TFPI and LUAD warrants further investigation. TFPI may influence LUAD through TF pathway, involvement in tumor progression, potential roles in drug resistance, and links to smoking-induced changes. Future research should focus on elucidating the mechanisms through which TFPI is involved in LUAD development, especially in the context of smoking-induced changes. These insights could pave the way for targeted therapeutic strategies. Exploring the specific role of TFPI in LUAD and its interactions with smoking-induced factors will necessitate comprehensive experimental and clinical studies. Moreover, it's worth noting that TFPI is currently under clinical trial for hematological malignancies like hemophilia.

RNASET2, a member of the T2 family of extracellular ribonucleases, has been associated with anti-tumor activities. Its overexpression inhibits the clonogenicity of ovarian cancer cells in vitro and suppresses tumorigenesis and metastasis in vivo [56]. However, our findings suggest that RNASET2 plays a role in promoting LUAD carcinogenesis. A meta-analysis revealed a correlation between RNASET2 and an elevated risk of lung cancer [24]. Its pro-cancer effects may involve promoting cancer cell migration, angiogenesis, and remodeling of the immune microenvironment [57]. Bioinformatics analysis of The Cancer Genome Atlas (TCGA) database indicates that the role of RNASET2 in tumor cells may be cancer-type-dependent and location-specific. For example, RNASET2 acts as a tumor suppressor in colorectal cancer, melanoma, non-Hodgkin's B-cell lymphoma, and acute lymphoblastic leukemia. However, it functions as an oncogene in renal cell carcinima and lung cancer [58]. In our research, we validated RNASET2 using a co-localization method. Although we did not find evidence of RNASET2 interacting with the targets of current LUAD drugs, it was the only biomarker validated in the FinnGen cohort, suggesting a higher likelihood of RANSET2 being a causal protein for LUAD. Future research could focus on elucidating the specific molecular pathways and genetic backgrounds associated with RNASET2 in different cancers.

VWC2 (Brorin) is a glycoprotein belonging to the Chordin family of secreted BMP regulators. Its expression is diminished in colorectal cancer and exhibits a negative correlation with tumor stage. VWC2 inhibits tumor cell growth both in vitro and in vivo [59]. Colonic adenocarcinoma displays the highest degree of DNA methylation in VWC2. Small ubiquitin-like modifier 1 (SUMO1)-mediated SUMOylation of DNA methyltransferase 1 (DNMT1) enhances its protein stability, promoting DNA methylation and subsequently downregulating VWC2. Currently, no studies have explored the association between VWC2 and LUAD. In our research, we established a link between VWC2 and reduced LUAD risk, confirming a causal effect through co-localization analysis. Additionally, we observed that higher levels of VWC2 were associated with leisure activities, suggesting that the protective effect of VWC2 on LUAD can be strengthened through such activities. Its potential role in LUAD involves a confirmed link to reduced risk and a proposed enhancement of protective effects through leisure activities. The mechanisms involve DNA methylation regulation, impacting VWC2 expression levels.

Tumor angiogenesis is a significant hallmark of cancer, and vascular endothelial growth factors (VEGFs) and their receptors play key roles in pathological angiogenesis in various tumors [60]. FLT1(VEGFR-1) is expressed on both endothelial and epithelial cells, binding with VEGF-A, VEGF-B, and placental growth factor (PGF) [61]. The mechanism of FLT1 in tumor cells remains unclear. Upregulated in colorectal cancer, FLT1 promotes epithelial-mesenchymal transition (EMT), enhancing invasiveness [62]. It also promotes proliferation, invasion, and metastasis of LUAD cells, with expression negatively correlated with survival time and recurrence-free survival rate in LUAD [63]. However, Dylan found that the variant allele (C) of the FLT1 SNP rs9582036 is associated with reduced FLT1 expression, consequently accelerating the recurrence of non-small cell lung cancer (NSCLC) through angiogenesis. One mechanism is that soluble FLT1 dominantly inhibits VEGFA by forming heterodimers with VEGFR-2, the primary receptor responsible for most proangiogenic effects of VEGFA [64]. Our results align with this, as the FLT1 protein variant offers protection against LUAD. Furthermore, PPI analysis revealed FLT1 interaction with EGFR, KRAS, ERBB2, and CD274 (PD-L1), all targeted by medications involving tyrosine kinase inhibitors and monoclonal antibodies. Consequently, FLT1 may represent a promising novel target for these anti-tumor drugs. Therapeutic agents targeting FLT1 have been well-developed and evaluated in phase II clinical trials.

In addition to the proteins mentioned above, our findings suggest that NTM has potential for both pharmacological and clinical applications. NTM is a glycoprotein belonging to the immunoglobulin superfamily and the IgLON family. Lower NTM gene expression was observed in LUAD compared to controls [65], a discovery confirmed by Oncomine and TCGA. However, our results indicate that NTM is associated with an increased risk of LUAD. PPI analysis has shown that NTM interacts with NTRK2, the targets of Larotrectinib and Entrectinib. This implies that NTM may influence LUAD by exerting its effects on NTRK.

There are four proteins not currently listed as druggable. The ALAD genetic polymorphism (rs1800435) is associated with reduced cancer mortality [66]. ALAD is significantly decreased in breast and hepatocellular carcinoma, and its reduced expression is correlated with an unfavorable prognosis in patients [67, 68]. Yh Yang et al. also found a significant association between ICAM5 and LUAD risk, suggesting its potential as a target for lung cancer [69, 70]. MDGA2 is a tumor suppressor in gastric carcinogenesis, and its hypermethylation is an independent prognostic factor in gastric cancer patients [71]. However, advanced-stage nasopharyngeal carcinoma patients exhibited higher MDGA2 expression levels compared to those in the early stage, aligning with our results [72]. Consistent with our findings, knockdown of PMM2 in renal cell carcinoma cells inhibited cancer cell migration and invasion, indicating that overexpression of PMM2 could promote malignancy [73]. However, there is currently no relevant research on ALAD, MDGA2, and PMM2 in relation to LUAD. Future research should focus on unraveling the specific molecular mechanisms of these proteins in lung cancer, exploring their potential as therapeutic targets, and developing drugs.

Our study utilized a two-sample MR and Bayesian co-localization analysis, identifying nine plasma proteins causally linked to LUAD. This underscores the efficacy of our approach in elucidating the fundamental mechanisms involved in the pathogenesis of LUAD. Our research has the potential to improve genetic-based screening methods for early-stage LUAD, providing valuable evidence for the development of screening and prevention strategies, particularly for individuals at high risk due to confirmed genetic factors. According to our study, personalized medical interventions targeting these proteins hold potential value in formulating prevention strategies, customizing screening plans, and intervening in genetic risks.

To further validate the functional role of the identified proteins, in future research, we will conduct in vitro experiments to investigate the effects of manipulating the identified proteins on LUAD cell lines. This involves accessing changes in cell proliferation, migration, or apotosis. Additionally, in vivo studies using animal models can be consisdered to evaluate the therapeutic efficacy of targeting these proteins.

To facilitate the transition to personalized medicine, we will take the following steps: First, we will investigate if the identified proteins can serve as reliable biomarkers for LUAD. This may involve analyzing patient samples to assess the correlation between protein levels and disease progression, treatment response, or patient outcomes. Second, we will explore the development of targeted therapies that specifically inhibit or modulate the activity of the identified proteins. This might include designing experiments to test the effectiveness of inhibitors, monoclonal antibodies, or gene therapies in suppressing LUAD cell growth or improving the sensitivity of cancer cells to existing treatments. Thrid, we will consider proposing clinical trials to evaluate the efficacy and safety of personalized treatments incorporating the targeted therapies based on the identified proteins. These trials could involve stratifying patients based on biomarker expression levels or genetic profiles to evaluate treatment responses in specific subgroups.

Our research findings have the potential to influence clinical guidelines and policies in several aspects. First, we identified potential biomarkers for LUAD, suggesting updates to screening guidelines based on correlations between specific proteins and the disease. Second, our work provides a basis for personalized prevention strategies for genetically at-risk populations, including customized screenings and interventions. Moreover, the study supports genetic counseling and patient education.

This study has several limitations. First, the LUAD GWAS data lack stratification based on specific subtypes, hindering stratified analyses. This limitation suggest potential for future research collaborations or consortia to collect more nuanced, stratified datasets. Second, caution is needed when interpreting the posterior probability of hypothesis 4 (PPH4) in colocalization, as a low PPH4 may not indicate the absence of evidence supporting colocalization, especially when PPH3 is also low due to limited statistical power [74]. Enhancing the power of existing colocalization methods through improved analytical estimation, fine-mapping methods, and explicit modeling of varying LD patterns across datasets could address this issue [74]. Third, a key assumption of MR is the “no horizontal pleiotropy” assumption. This assumes that the IV used for MR exclusively influences the outcome through the exposure. Horizontal pleiotropy occurs when the variant affects traits beyond the exposure pathway or directly impacts the outcome [33]. Despite excluding trans-associated loci, conducting Steiger filtering, and sensitivity analyses in our MR study, fully eliminating horizontal pleiotropy and confounding bias remains challenging. Acknowledge these limitations is crucial due to their potential impact on the study’s conclusions. Fourth, weak instrument bias in MR remains a challenge, which can lead to inaccurate estimates and reduced statistical power. To mitigate this, we selected genetic instruments with established associations with the exposure variable and employed various statistical methods and sensitivity analyses. However, some level of bias may still exist. Fifth, caution is needed when generalizing our findings, as both the pQTLs and GWAS data primarily originated from individuals of European ancestry. Extending these findings to other ethnicities requires further validation. Finally, our study revealed limited genetic prediction of TFPI and VWC2 mediated by smoking and leisure activities, indicating a need for additional research to explore other potential mediators.

In conclusion, our genetic association studies suggest a causality between genetically determined plasma proteins and LUAD. The identified proteins, particularly RNASET2, TFPI, VWC2, FLT1, and NTM, show promise as potential therapeutic targets for LUAD. Further research is needed to elucidate the mechanisms of these candidate proteins in LUAD.

### Supplementary Information

**Additional file 1:** **Figure S1.** Study flame chart of the Mendelian randomization study. We identified SNPs associated with plasma proteins. Various Mendelian Randomization (MR) approaches were used to access the causality of plasma proteins for LUAD. When only one pQTL was available for a protein, we applied the Wald ratio method. If two or more pQTLs were available, we used the inverse-variance-weighted (IVW), MR Egger, weighted median, weighted mode, and simple mode. If only two SNPs were found, only the IVW method was employed. Then, sensitivity analyses were conducted to detect underlying pleiotropy and heterogeneity. The Cochran Q test (*P* < 0.05) from the IVW approach was used to identify potential horizontal pleiotropy. The intercept obtained from the MR-Egger regression indicated directional pleiotropy (*P* < 0.05). Additionally, MR-PRESSO was used to assess horizontal pleiotropy. pQTLs: protein quantitative trait locus; SNPs: single nucleotide polymorphisms; LUAD: lung adenocarcinoma; GWAS: genome-wide association study; MR-PRESSO: MR Pleiotropy Residual Sum and Outlier. **Figure S2.** Standard MR plots for proteins and risk of LUAD. MR analysis identified plasma proteins associated with LUAD risk. The different regression lines indicated the effect sizes as calculated by different MR tests (methods). MR analysis of plasma proteins for ALAD (**a**), FLT1 (**b**), ICAM5 (**c**), MDGA2 (**d**), NTM (**e**), PMM2 (**f**), RNASET2 (**g**), and VWC2 (**h**), respectively. ALAD: aminolevulinate dehydratase; FLT1: Fms related receptor tyrosine kinase 1; ICAM5: intercellular adhesion molecule 5; MDGA2: MAM domain containing glycosylphosphatidylinositol anchor 2; NTM: neurotrimin; PMM2: phosphomannomutase 2; RNASET2: ribonuclease T2; VWC2: von willebrand factor C domain containing 2. MR: Mendelian randomization; LUAD: lung adenocarcinoma. **Figure S3.** External validation of the causal relationship between six potential causal proteins and LUAD through MR analysis. The squares were the causal estimates on the OR scale, and the whiskers represented the 95% CI for these ORs. nSNPs: number of SNPs used for the estimation of the causal effects in this plot. OR for increased risk of LUAD were expressed as per SD increase in plasma protein levels. *P* values were determined from the inverse-variance-weighted (IVW) MR method. OR: odds ratio; CI: confidence interval; MR: LUAD: lung adenocarcinoma; SD: standard deviation. **Figure S4.** Bidirectional MR analysis for LUAD on levels of nine potential causal proteins. OR for increased risk of LUAD were expressed as per SD increase in plasma protein levels. OR: odds ratio; CI: confidence interval; MR: Mendelian randomization; LUAD: lung adenocarcinoma; SD: standard deviation. **Figure S5.** Colocalization plots of pQTLs and genetic associations of LUAD. Colocalization analysis of plasma proteins for ALAD (**a**), FLT1 (**b**), ICAM5 (**c**), MDGA2 (**d**), NTM (**e**), PMM2 (**f**), PTGFRN (**g**), TFPI (**i**), and VWC2 (**j**), respectively. Diamond purple points represented the SNP that with the minimal sum of *P* value in corresponded protein GWAS and LUAD GWAS. pQTL: protein quantitative trait loci; LUAD: lung adenocarcinoma; SNP: single nucleotide polymorphism; GWAS: genome-wide association studies. **Figure S6.** Protein-protein interaction network among the causal proteins and current lung adenocarcinoma medications targets.

**Additional file 2.** **Table S1.** Genetic instruments of plasma proteins for MR analysis. MR: Mendelian randomization; SNP: single nucleotide polymorphism; chr: chromosome; pos: position; eaf: effect allele frequency; se: standard error. **Table S2.** MR results for plasma proteins significantly associated with LUAD after Bonferroni correction. MR: Mendelian randomization; LUAD: lung adenocarcinoma; SNP: single nucleotide polymorphism; OR: odds ratio; CI: confidence interval; PVE: proportion of variance explained. **Table S3.** MR results of proteome and LUAD. MR: Mendelian randomization; LUAD: lung adenocarcinoma; SNP: single nucleotide polymorphism; nSNPs: number of SNPs; se: standard error; OR: odds ratio; CI: confidence interval; IVW:inverse-variance-weighted. **Table S4.** Steiger filtering and reverse MR results of LUAD as exposure and proteome as outcome. MR: Mendelian randomization; LUAD: lung adenocarcinoma; SNP: single nucleotide polymorphism; nSNPs: number of SNPs; OR: odds ratio; CI: confidence interval; IVW:inversevariance-weighted. **Table S5.** Bayesian co-localization analysis on nine potential causal proteins. Druggable genes were divided into three tiers, including targets of approved drugs and drugs in clinical development (tier 1), proteins closely related to drug targets or with associated drug-like compounds (tier 2), and extracellular proteins and members of key drug-target families (tier 3). SNP: single nucleotide polymorphism. **Table S6.** Previously-reported genome-wide significant association of SNPs as genetic instruments of three potential causal proteins. SNP: single nucleotide polymorphism; chr: chromosome; se: standard error; N_samples: number of samples; N_cases: number of cases; N_controls: number of controls. **Table S7.** MR results of risk factors versus LUAD (GWAS from European population). MR: Mendelian randomization; LUAD: lung adenocarcinoma; GWAS: genome-wide association study; SNP: single nucleotide polymorphism; nSNPs: number of SNPs; se: standard error; OR: odds ratio; CI: confidence interval; IVW:inverse-variance-weighted. **Table S8.** MR results of proteome and LUAD risk factors. MR: Mendelian randomization; LUAD: lung adenocarcinoma; SNP: single nucleotide polymorphism; nSNPs: number of SNPs; se: standard error; OR: odds ratio; CI: confidence interval; IVW:inverse-variance-weighted; COPD: chronic obstructive pulmonary disease. **Table S9.** LUAD mediation results for protein targets on LUAD via risk factors. LUAD: lung adenocarcinoma. **Table S10.** Summary of druggability and drug development for LUAD associated with plasma proteins through MR analysis. Druggable genes were divided into three tiers, including targets of approved drugs and drugs in clinical development (tier 1), proteins closely related to drug targets or with associated drug-like compounds (tier 2), and extracellular proteins and members of key drug-target families (tier 3). LUAD: lung adenocarcinoma; NSCLC: non-small cell lung cancer.

## Data Availability

Summay data used for this study can be accessed through the following links: plasma protein, http://ukb-ppp.gwas.eu; lung adenocarcinoma, https://gwas.mrcieu.ac.uk/datasets/ieu-a-984/, https://storage.googleapis.com/finngen-public-datar9/summary_stats/finngen_R9_C3_NSCLC_ADENO_EXALLC.gz; Age of smoking initiation, https://gwas.mrcieu.ac.uk/datasets/ieu-b-24/; Number of cigarettes smoked daily, https://gwas.mrcieu.ac.uk/datasets/ukb-b-6019/; Pack years of smoking, https://gwas.mrcieu.ac.uk/datasets/ukb-b-10831/; Maternal smoking around birth, https://gwas.mrcieu.ac.uk/datasets/ukb-b-17685/; Any parental history of lung cancer, https://gwas.mrcieu.ac.uk/datasets/ebi-a-GCST90013922/; Average weekly beer intake, https://gwas.mrcieu.ac.uk/datasets/ukb-b-5174/; Leisure activities, https://gwas.mrcieu.ac.uk/datasets/ukb-b-4667/; COPD, https://gwas.mrcieu.ac.uk/datasets/ebi-a-GCST90018807/.
