# Supplementary material for: Systematic proteome-wide Mendelian randomization using the human plasma proteome to identify therapeutic targets for lung adenocarcinoma
Source: J Transl Med. 2024 Apr 4;22:330. doi: 10.1186/s12967-024-04919-z (PMC10993587; doi:10.1186/s12967-024-04919-z)
Supplement: Supplementary file 1 — Additional file 1: Figure S1. Study flame chart of the Mendelian randomization study. We identified SNPs associated with plasma proteins. Various Mendelian Randomization (MR) approaches were used to access the causality of plasma proteins for LUAD. When only one pQTL was available for a protein, we applied the Wald ratio method. If two or more pQTLs were available, we used the inverse-variance-weighted (IVW), MR Egger, weighted median, weighted mode, and simple mode. If only two SNPs were found, only the IVW method was employed. Then, sensitivity analyses were conducted to detect underlying pleiotropy and heterogeneity. The Cochran Q test (P < 0.05) from the IVW approach was used to identify potential horizontal pleiotropy. The intercept obtained from the MR-Egger regression indicated directional pleiotropy (P < 0.05). Additionally, MR-PRESSO was used to assess horizontal pleiotropy. pQTLs: protein quantitative trait locus; SNPs: single nucleotide polymorphisms; LUAD: lung adenocarcinoma; GWAS: genome-wide association study; MR-PRESSO: MR Pleiotropy Residual Sum and Outlier. Figure S2. Standard MR plots for proteins and risk of LUAD. MR analysis identified plasma proteins associated with LUAD risk. The different regression lines indicated the effect sizes as calculated by different MR tests (methods). MR analysis of plasma proteins for ALAD (a), FLT1 (b), ICAM5 (c), MDGA2 (d), NTM (e), PMM2 (f), RNASET2 (g), and VWC2 (h), respectively. ALAD: aminolevulinate dehydratase; FLT1: Fms related receptor tyrosine kinase 1; ICAM5: intercellular adhesion molecule 5; MDGA2: MAM domain containing glycosylphosphatidylinositol anchor 2; NTM: neurotrimin; PMM2: phosphomannomutase 2; RNASET2: ribonuclease T2; VWC2: von willebrand factor C domain containing 2. MR: Mendelian randomization; LUAD: lung adenocarcinoma. Figure S3. External validation of the causal relationship between six potential causal proteins and LUAD through MR analysis. The squares were the causal estimates on the [file 12967_2024_4919_MOESM1_ESM.docx]

Systematic proteome-wide Mendelian randomization using the human plasma proteome to identify therapeutic targets for lung adenocarcinoma

**Figure S1 Study flame chart of the Mendelian randomization study.**

**Figure S2 Standard MR plots for proteins and risk of LUAD.**

**Figure S3 External validation of the causal relationship between six potential causal proteins and LUAD through MR analysis.**

**Figure S4 Bidirectional MR analysis for LUAD on levels of nine potential causal proteins.**

**Figure S5 Colocalization plots of pQTLs and genetic associations of LUAD.**

**Figure S6 Protein-protein interaction network among the causal proteins and current lung adenocarcinoma medications targets.**


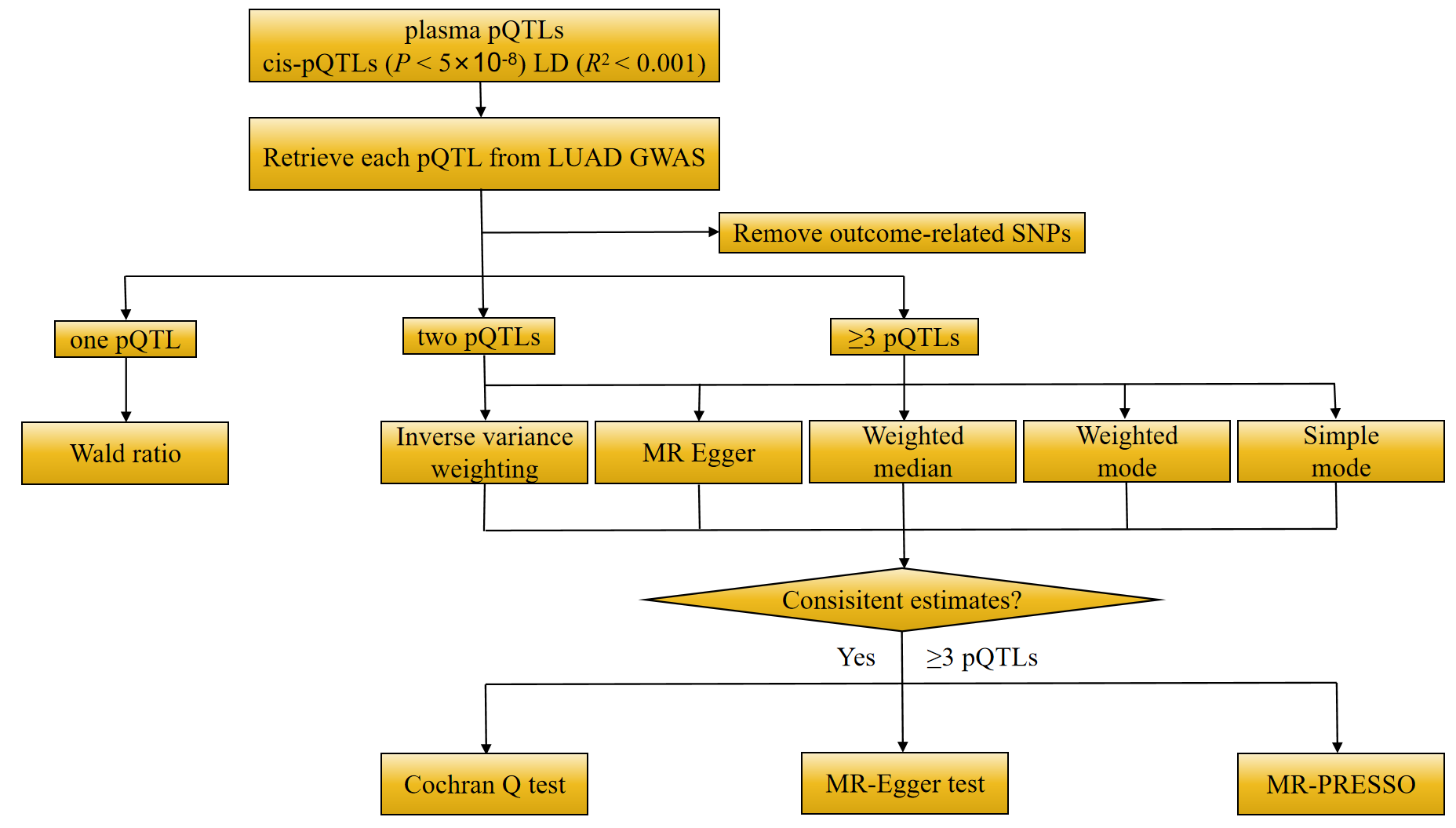


**Figure S1 Study flame chart of the Mendelian randomization study.** We identified SNPs associated with plasma proteins. Various Mendelian Randomization (MR) approaches were used to access the causality of plasma proteins for LUAD. When only one pQTL was available for a protein, we applied the Wald ratio method. If two or more pQTLs were available, we used the inverse-variance-weighted (IVW), MR Egger, weighted median, weighted mode, and simple mode. If only two SNPs were found, only the IVW method was employed. Then, sensitivity analyses were conducted to detect underlying pleiotropy and heterogeneity. The Cochran Q test (*P* < 0.05) from the IVW approach was used to identify potential horizontal pleiotropy. The intercept obtained from the MR-Egger regression indicated directional pleiotropy (*P* < 0.05). Additionally, MR-PRESSO was used to assess horizontal pleiotropy. pQTLs: protein quantitative trait locus; SNPs: single nucleotide polymorphisms; LUAD: lung adenocarcinoma; GWAS: genome-wide association study; MR-PRESSO: MR Pleiotropy Residual Sum and Outlier.

(a)ALAD (b) FLT1


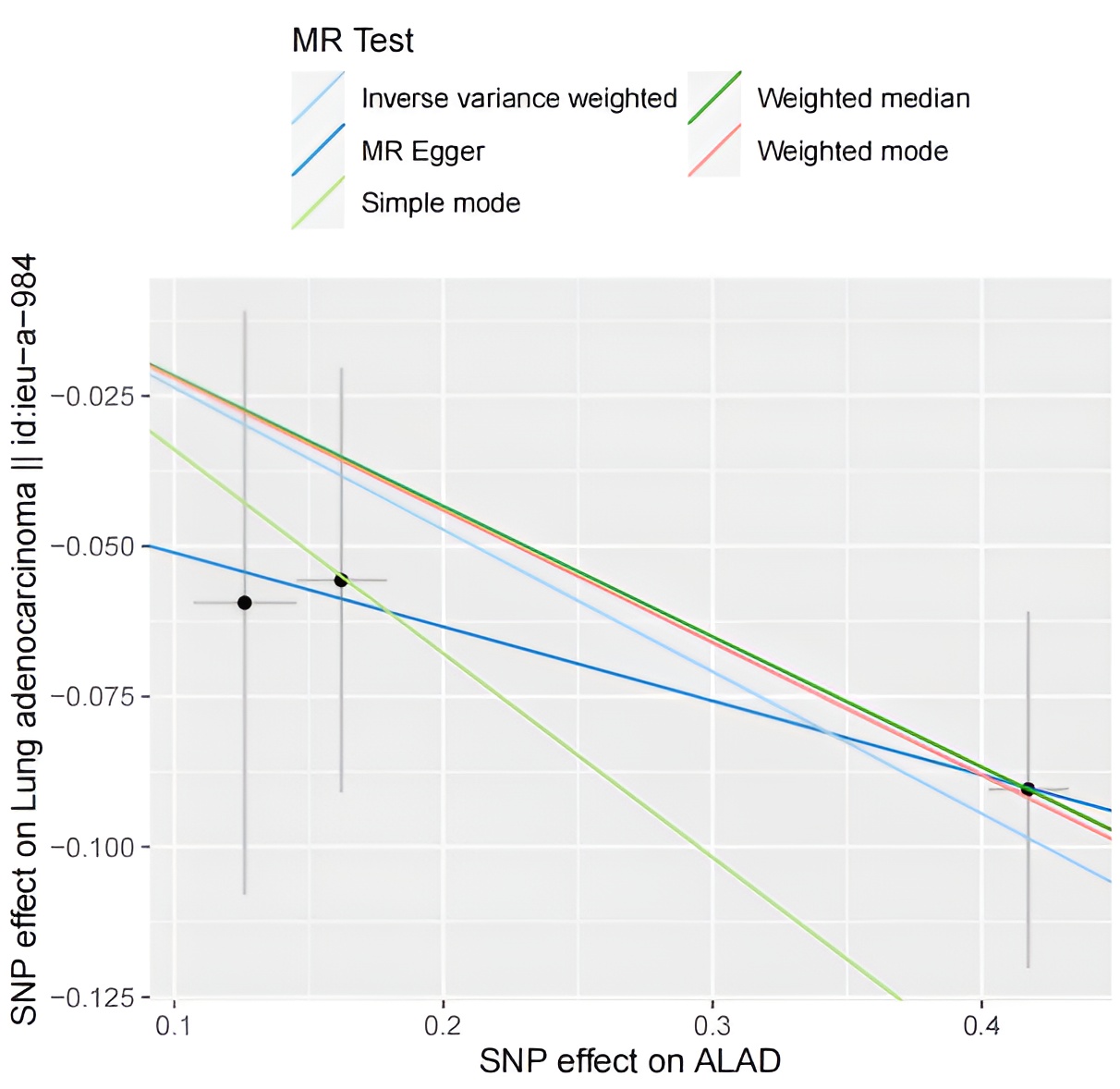

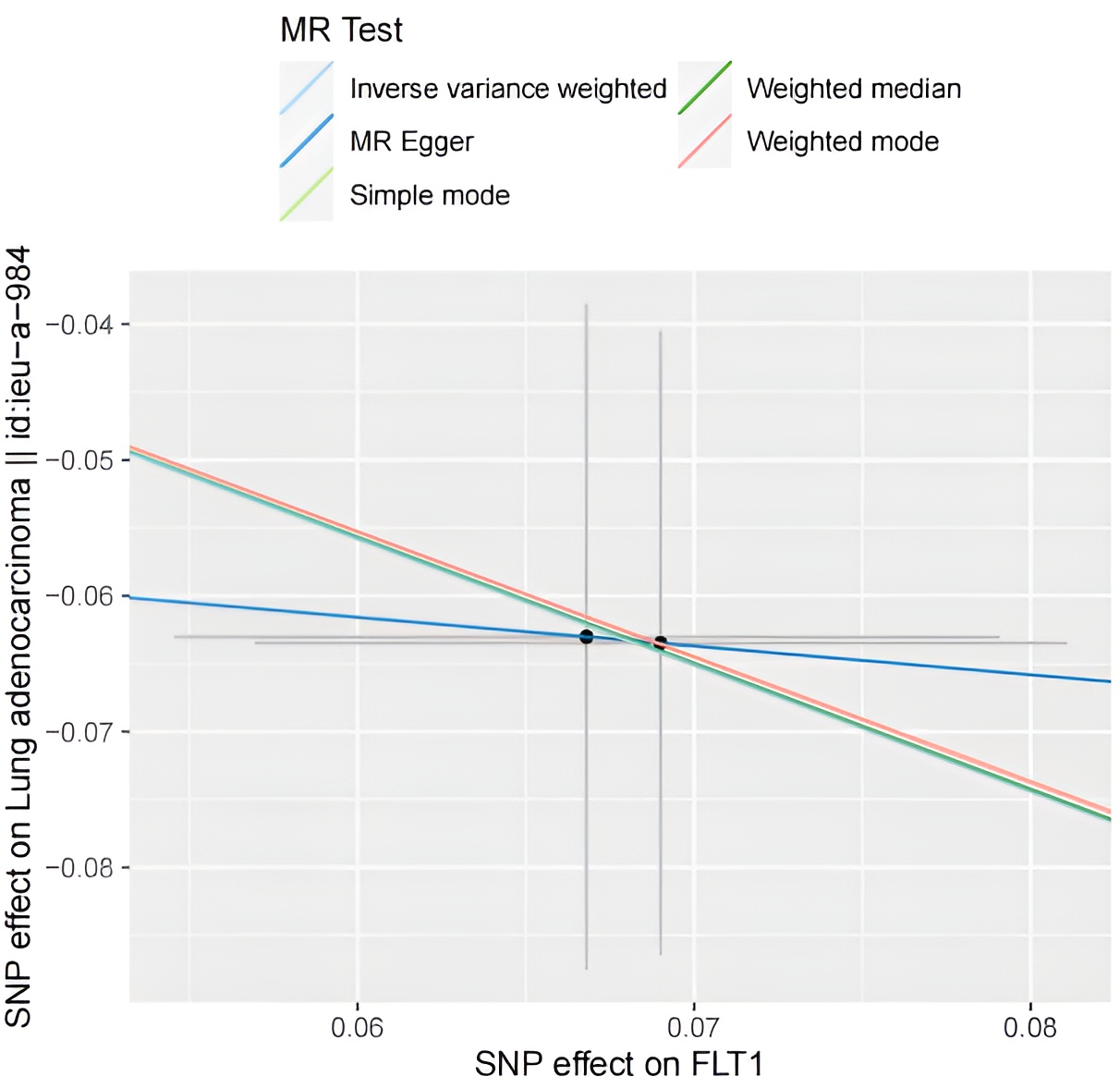


(c) ICAM5 (d) MDGA2


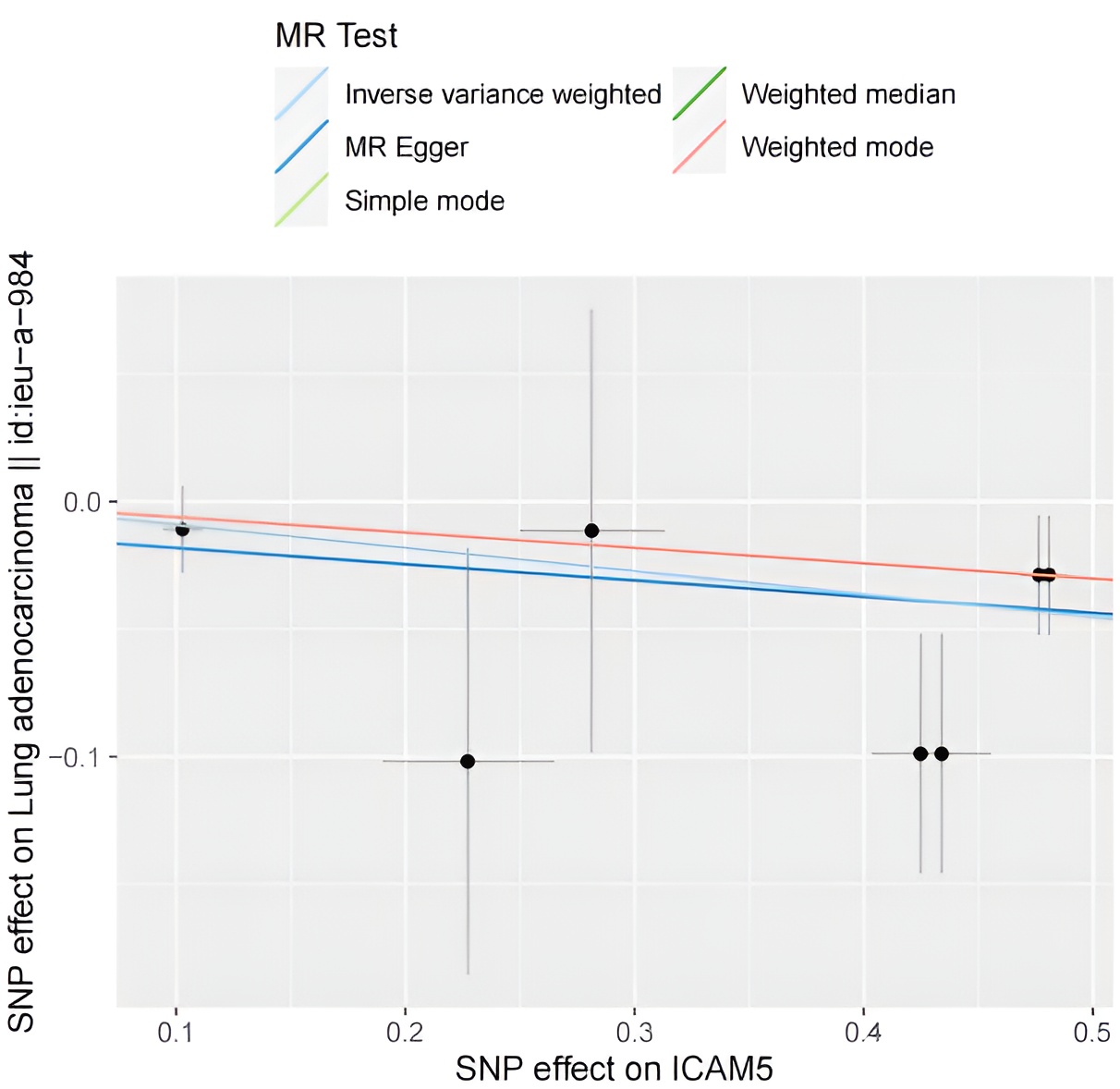

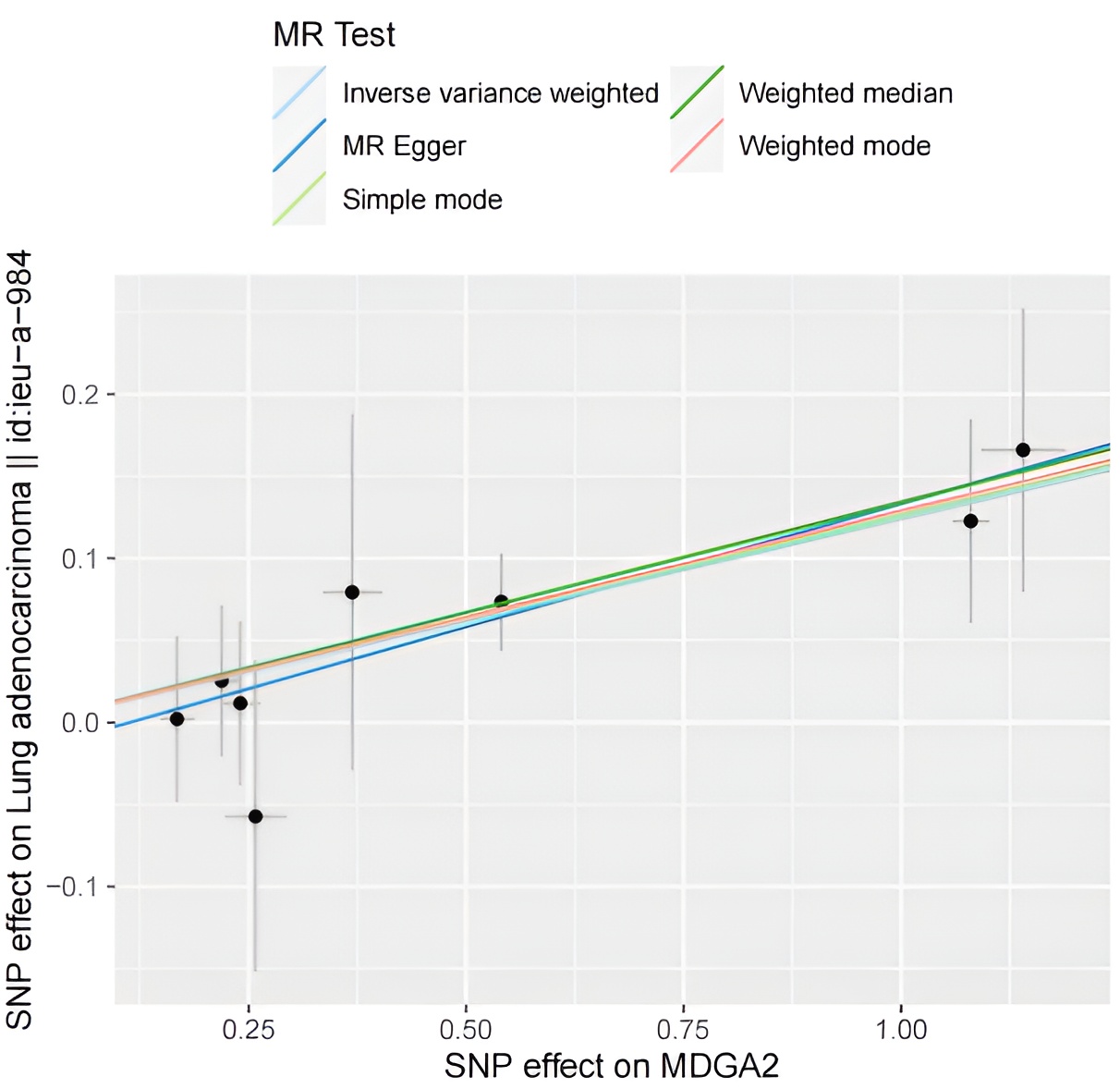


(e) NTM (f) PMM2


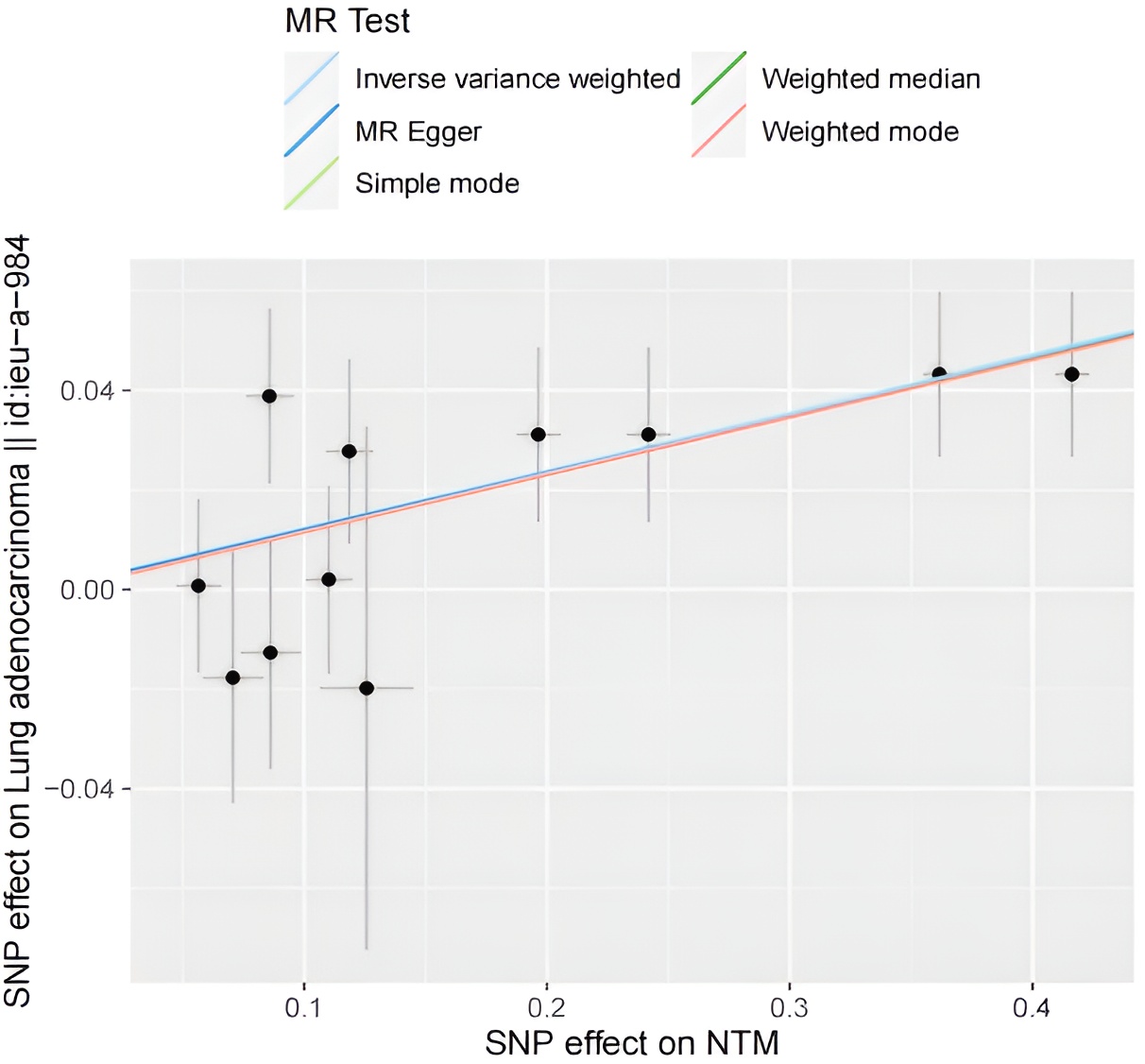

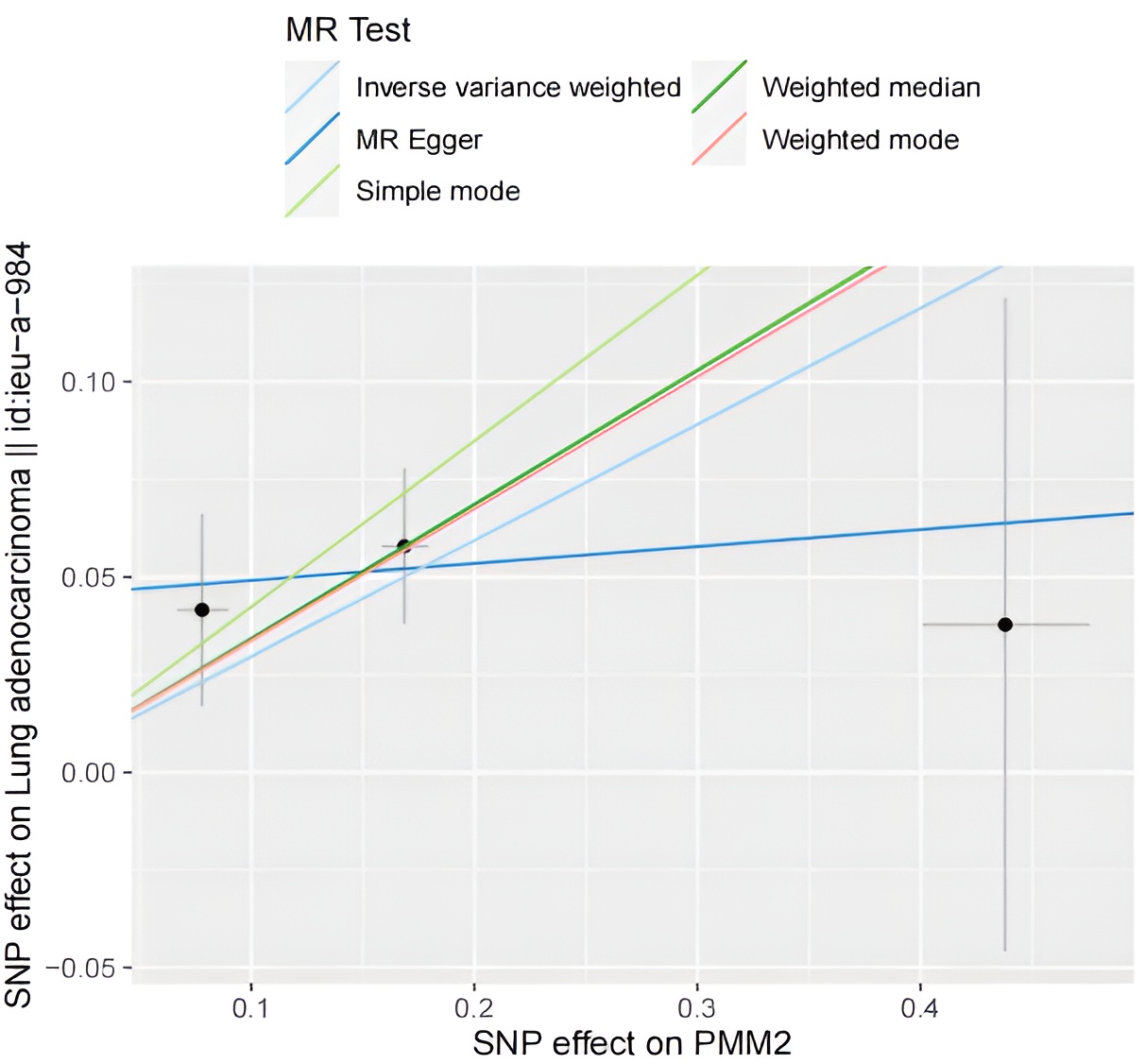


(g) RNASET2 (h)VWC2


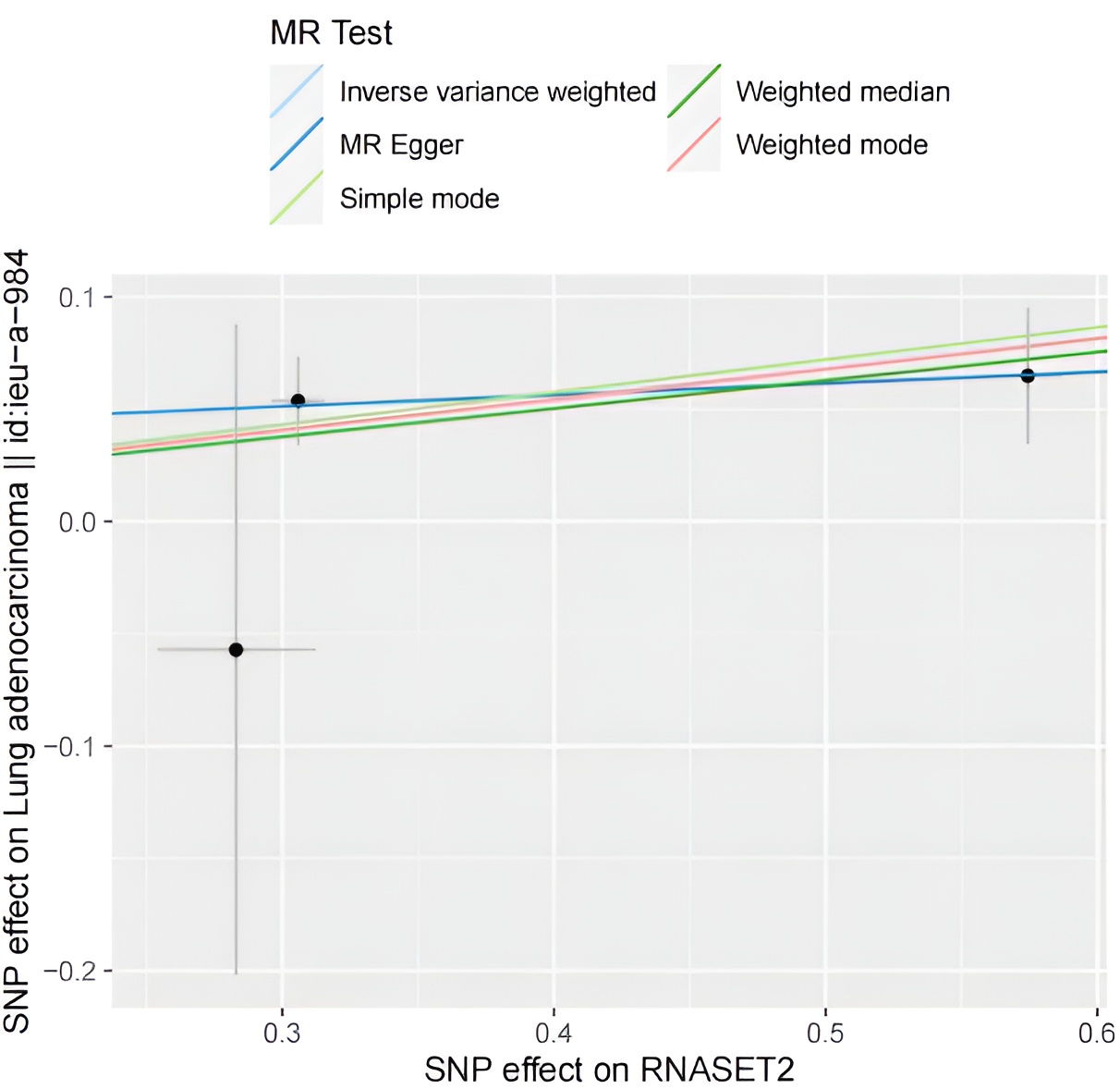

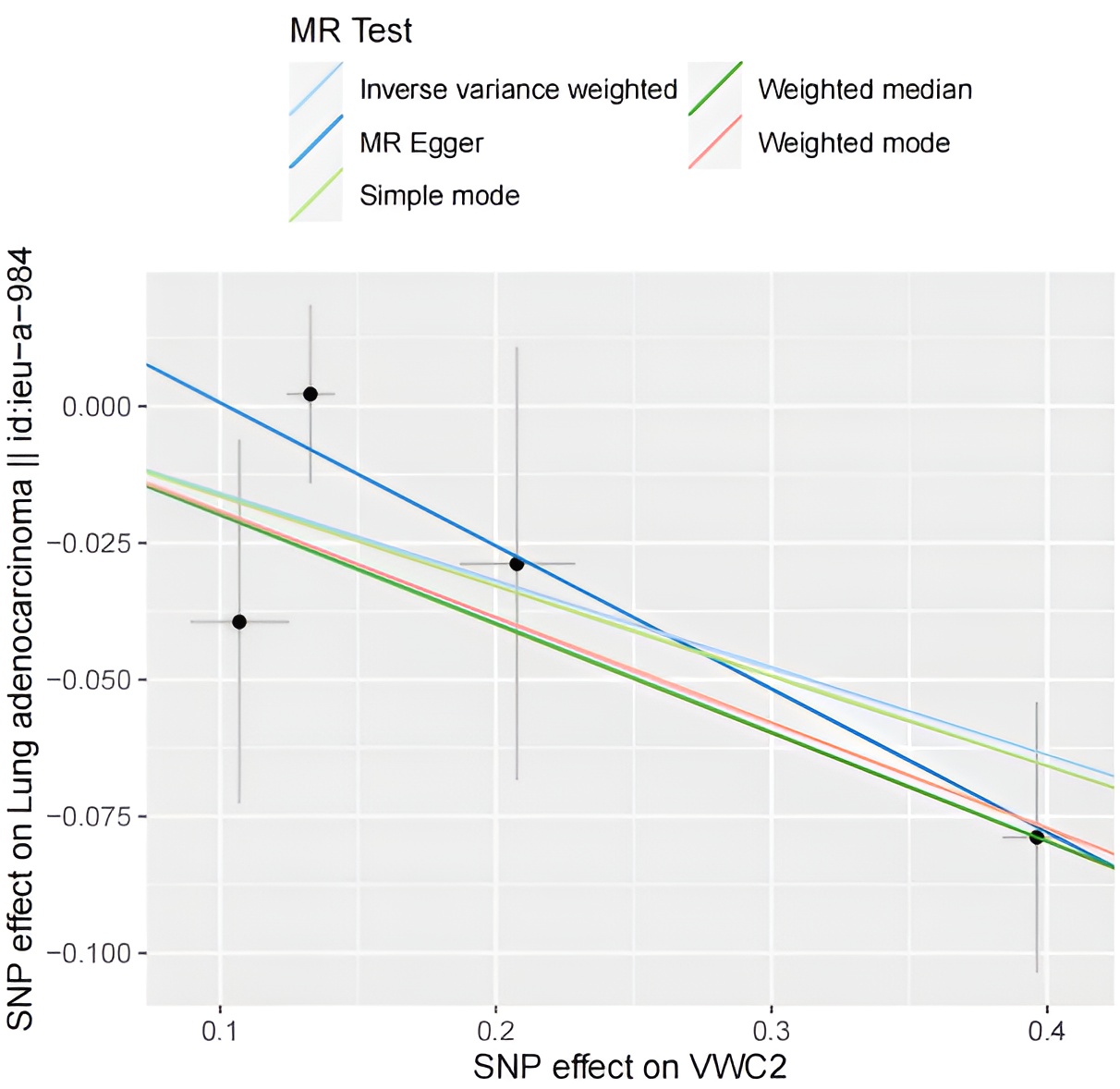


**Figure S2 Standard MR plots for proteins and risk of LUAD.** MR analysis identified plasma proteins associated with LUAD risk. The different regression lines indicated the effect sizes as calculated by different MR tests (methods). MR analysis of plasma proteins for ALAD (a), FLT1 (b) , ICAM5 (c), MDGA2 (d), NTM (e), PMM2 (f), RNASET2 (g), and VWC2 (h), respectively. Abbreviations: ALAD = aminolevulinate dehydratase; FLT1 = Fms related receptor tyrosine kinase 1; ICAM5 = intercellular adhesion molecule 5; MDGA2 = MAM domain containing glycosylphosphatidylinositol anchor 2; NTM= neurotrimin; PMM2 = phosphomannomutase 2; RNASET2= ribonuclease T2; VWC2= von willebrand factor C domain containing 2. MR: Mendelian randomization; LUAD: lung adenocarcinoma.


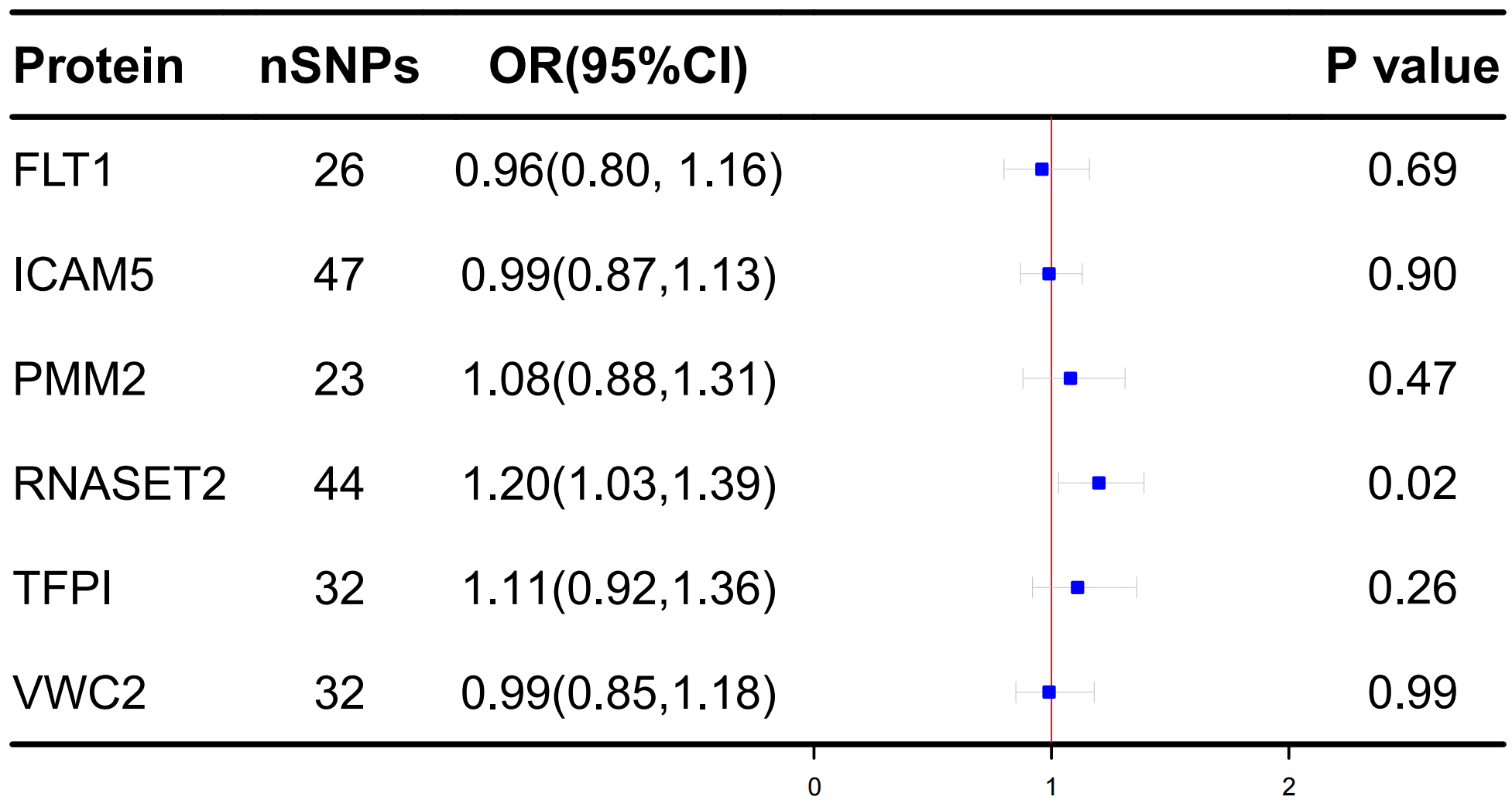


**Figure S3 External validation of the causal relationship between six potential causal proteins and LUAD through MR analysis.** The squares were the causal estimates on the OR scale, and the whiskers represented the 95% CI for these ORs. nSNPs: number of SNPs used for the estimation of the causal effects in this plot. OR for increased risk of LUAD were expressed as per SD increase in plasma protein levels. *P* values were determined from the inverse-variance-weighted (IVW) MR method. OR: odds ratio; CI: confidence interval; MR: LUAD: lung adenocarcinoma; SD: standard deviation.


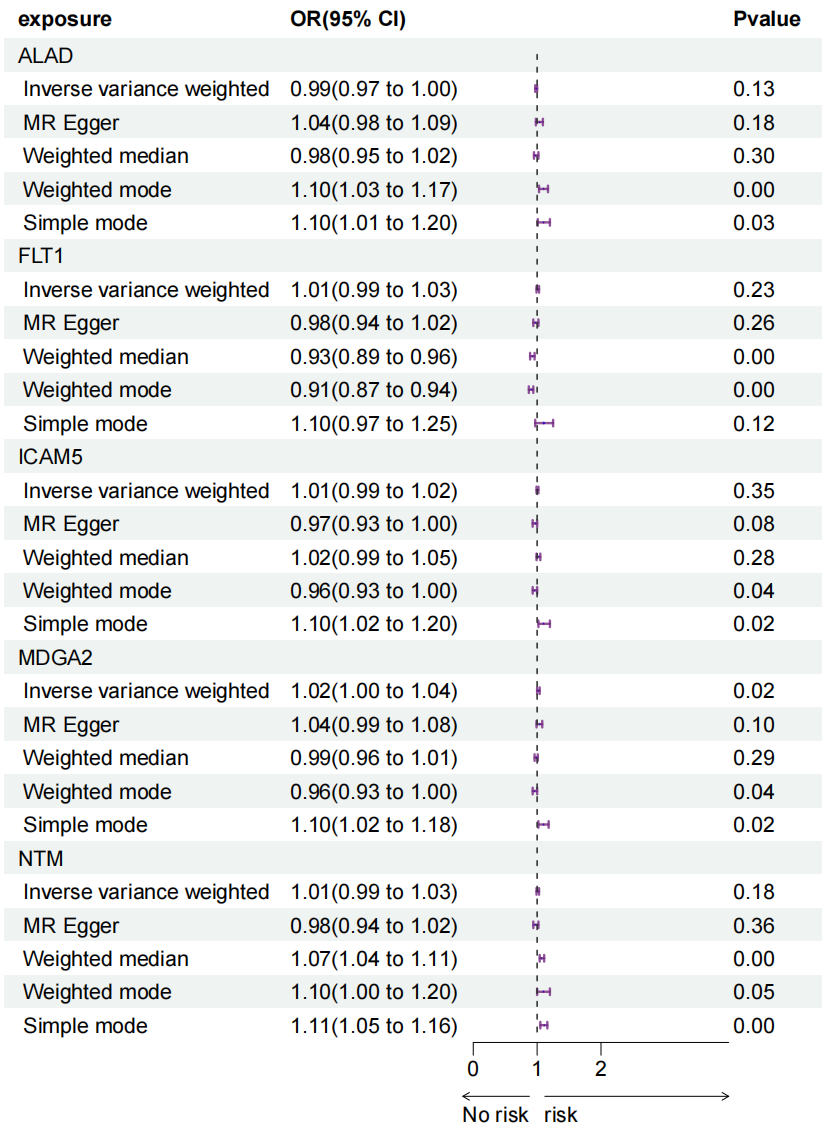

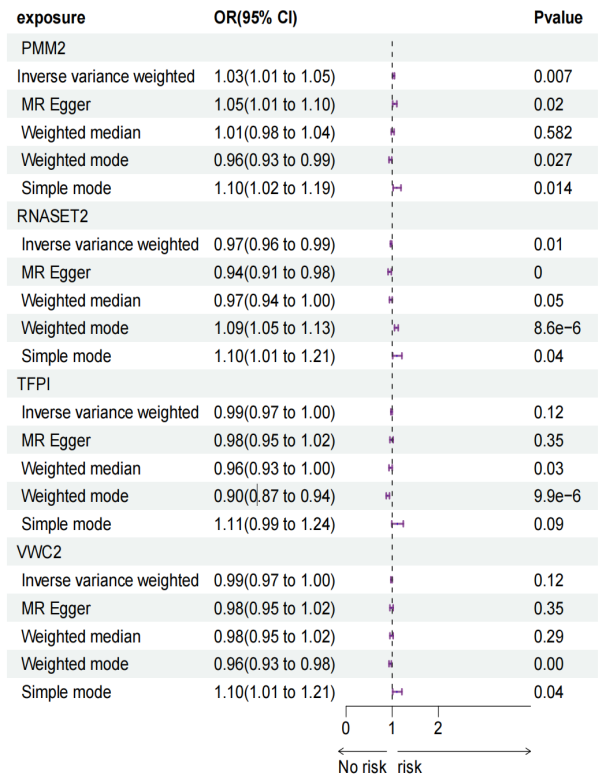


**Figure S4 Bidirectional MR analysis for LUAD on levels of nine potential causal proteins.** OR for increased risk of LUAD were expressed as per SD increase in plasma protein levels. OR: odds ratio; CI: confidence interval; MR: Mendelian randomization; LUAD: lung adenocarcinoma; SD: standard deviation.

(a)ALAD (b) FLT1


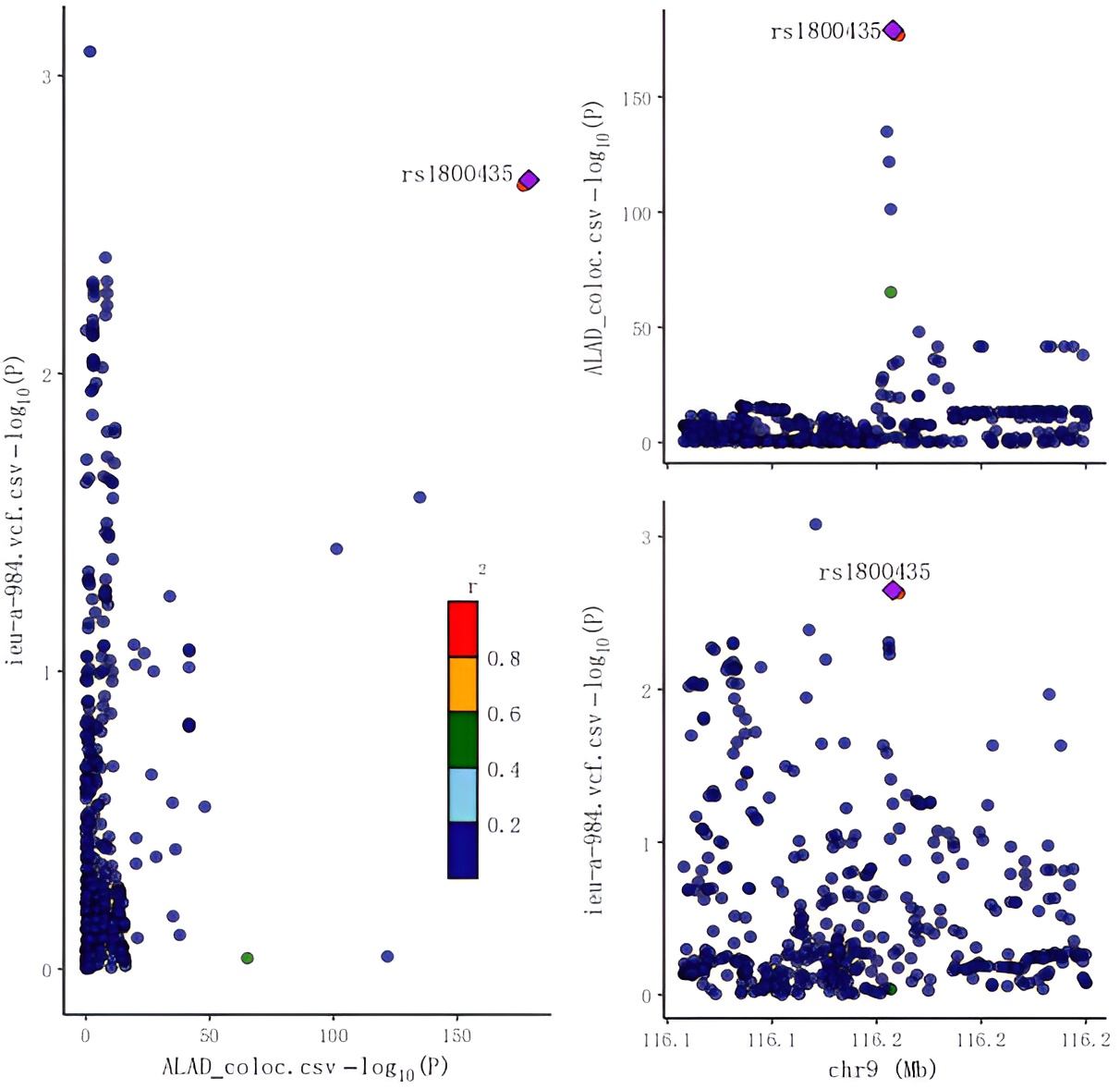

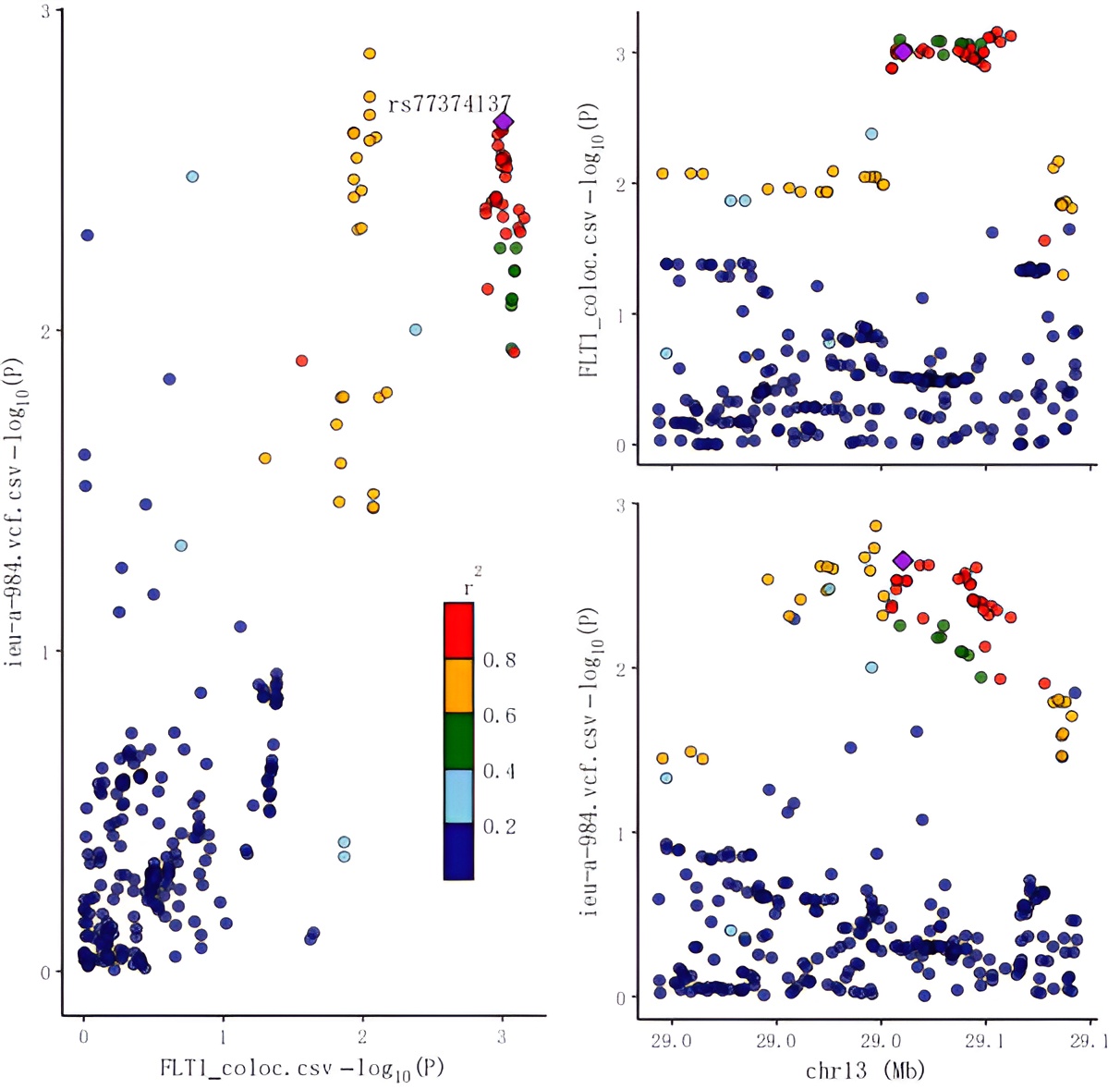


(c) ICAM5 (d) MDGA2


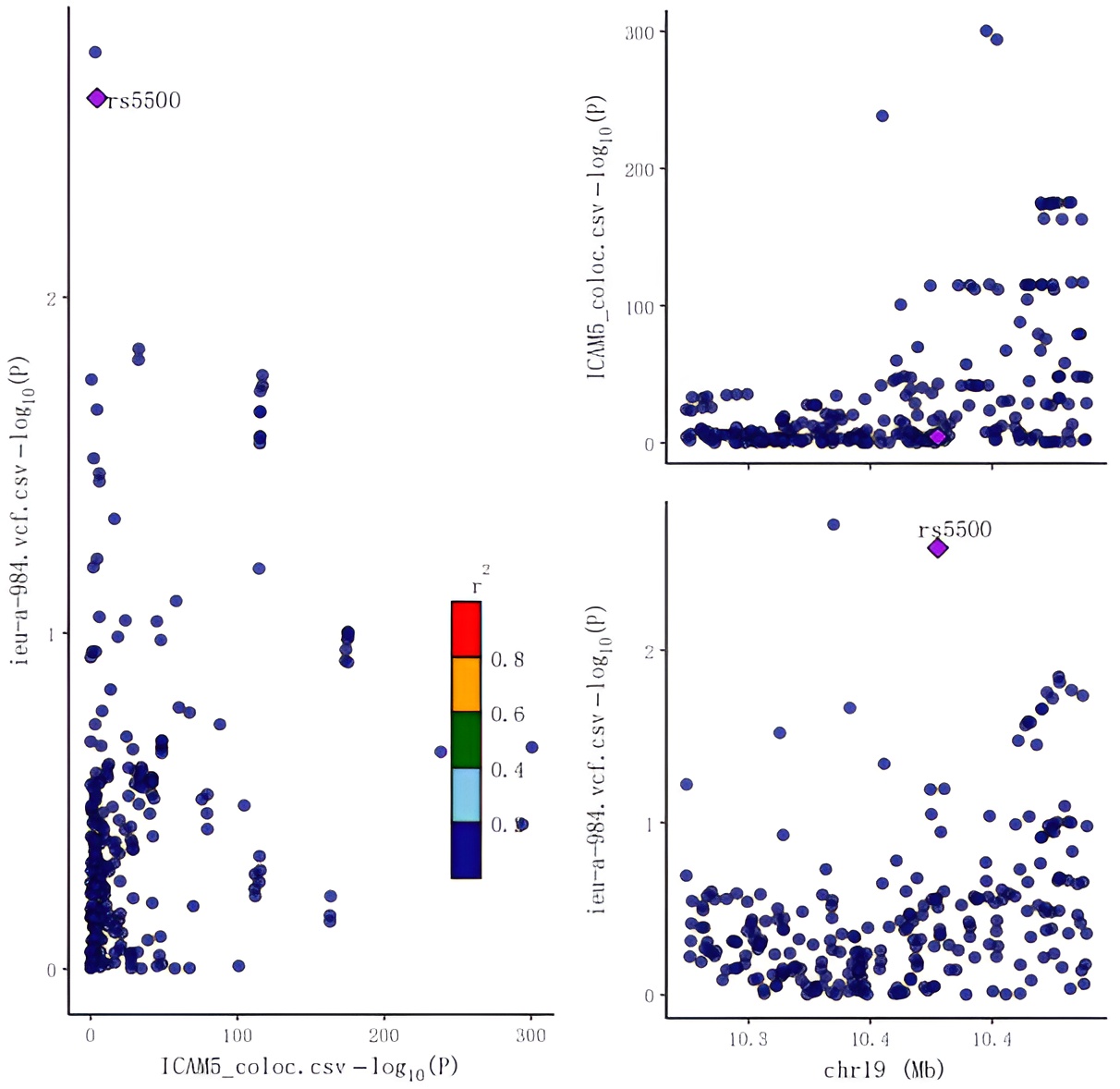

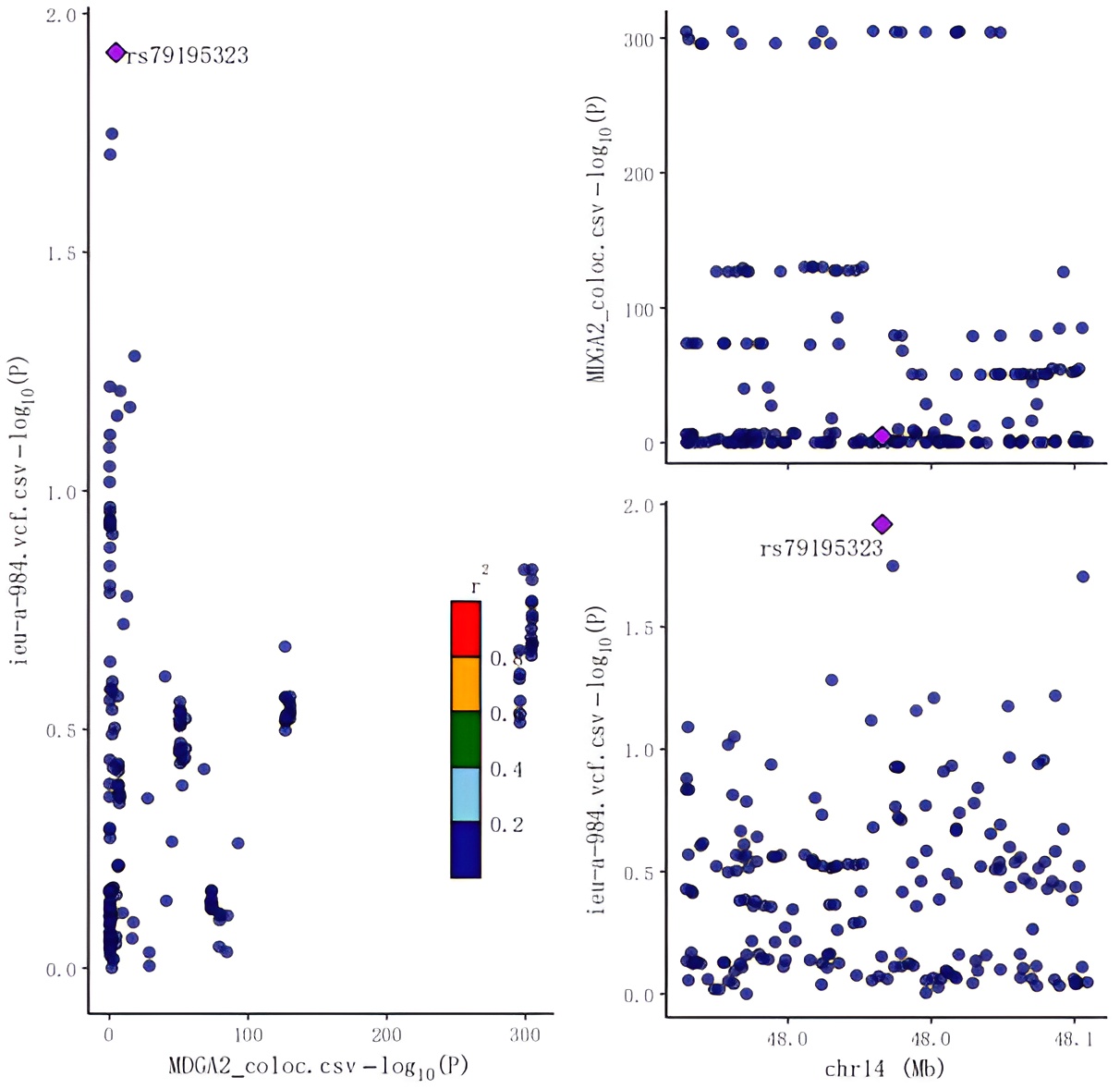


(e) NTM (f) PMM2


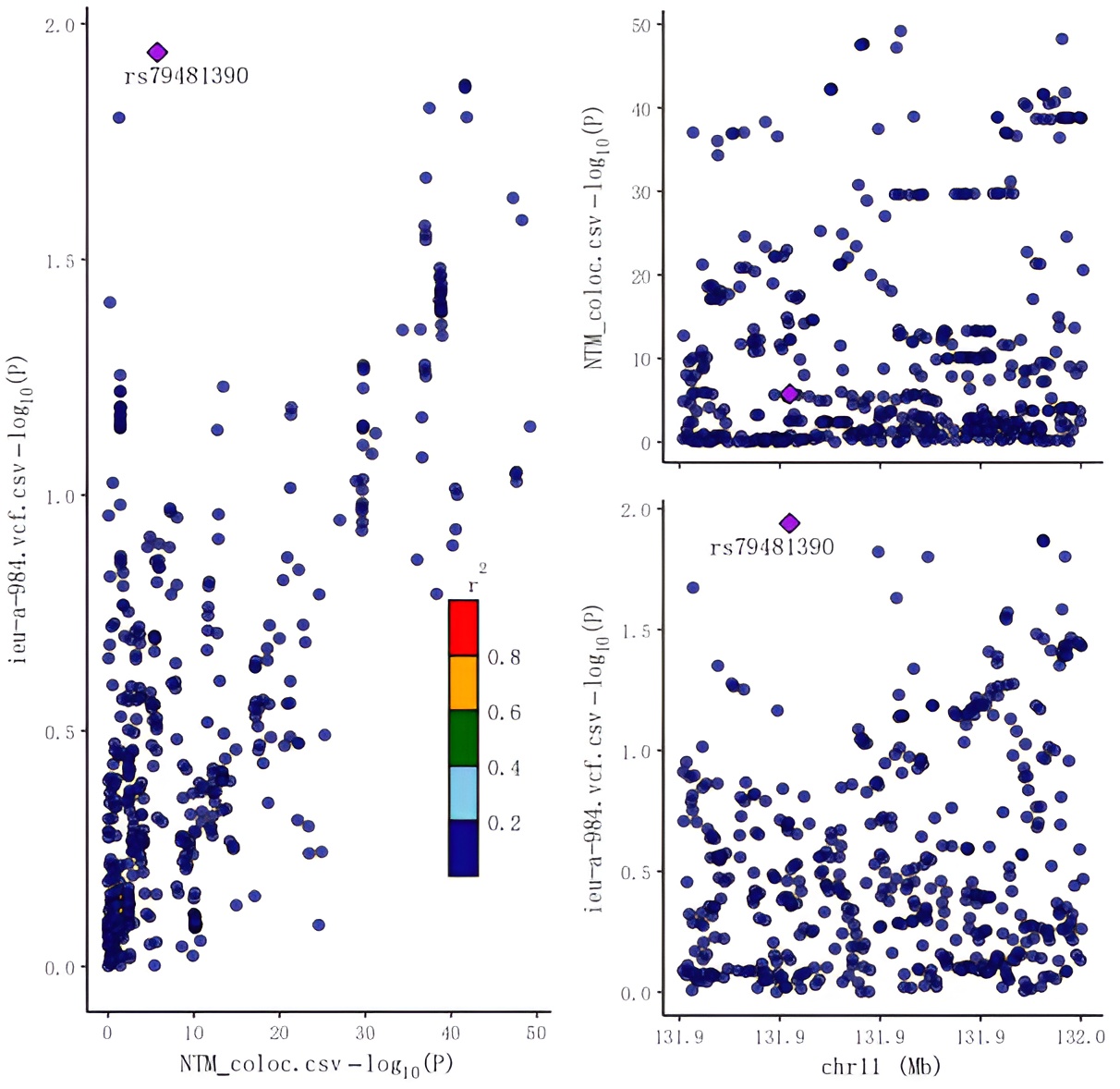

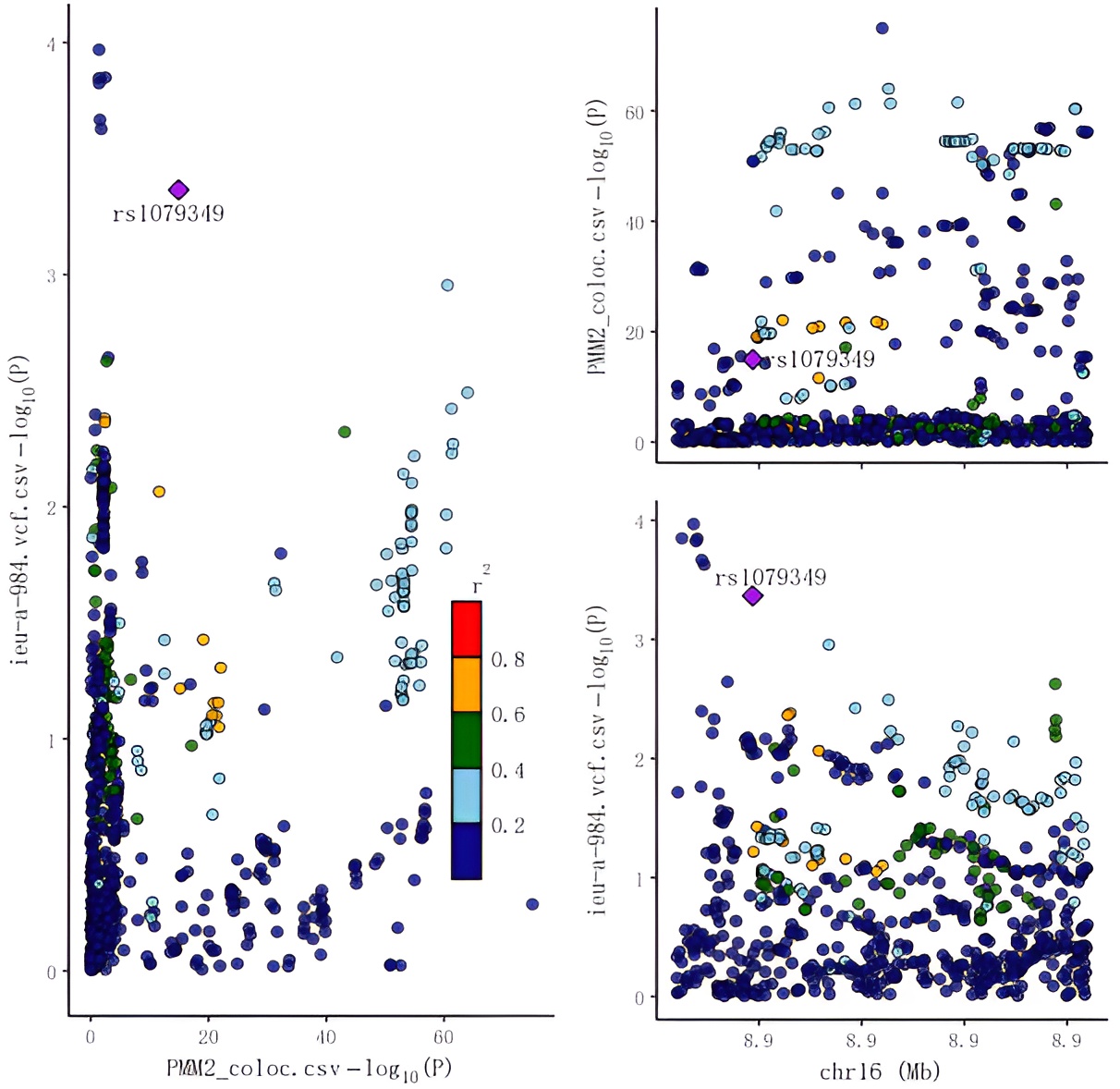


(g) RNASET2 (h) TFPI


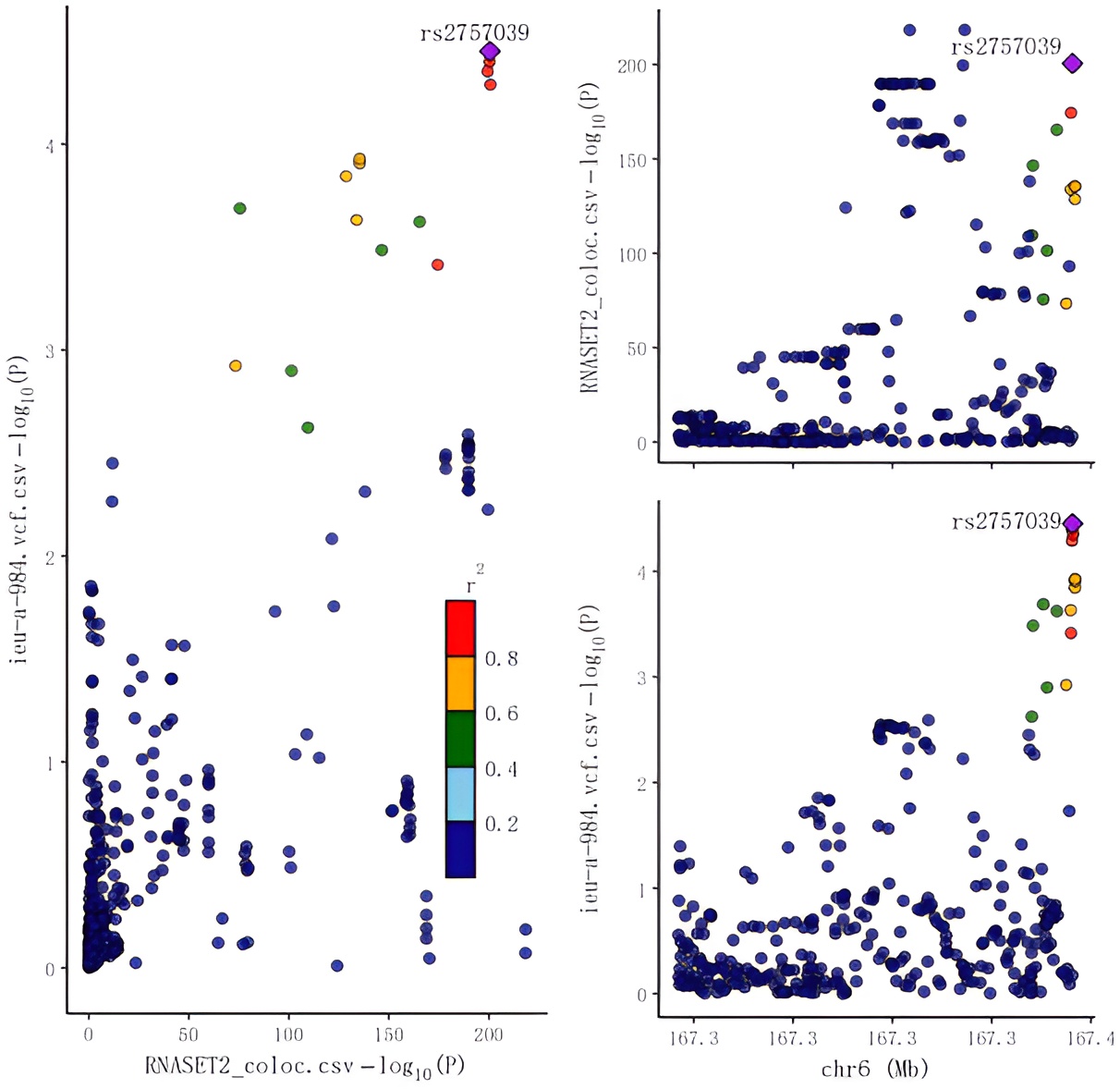

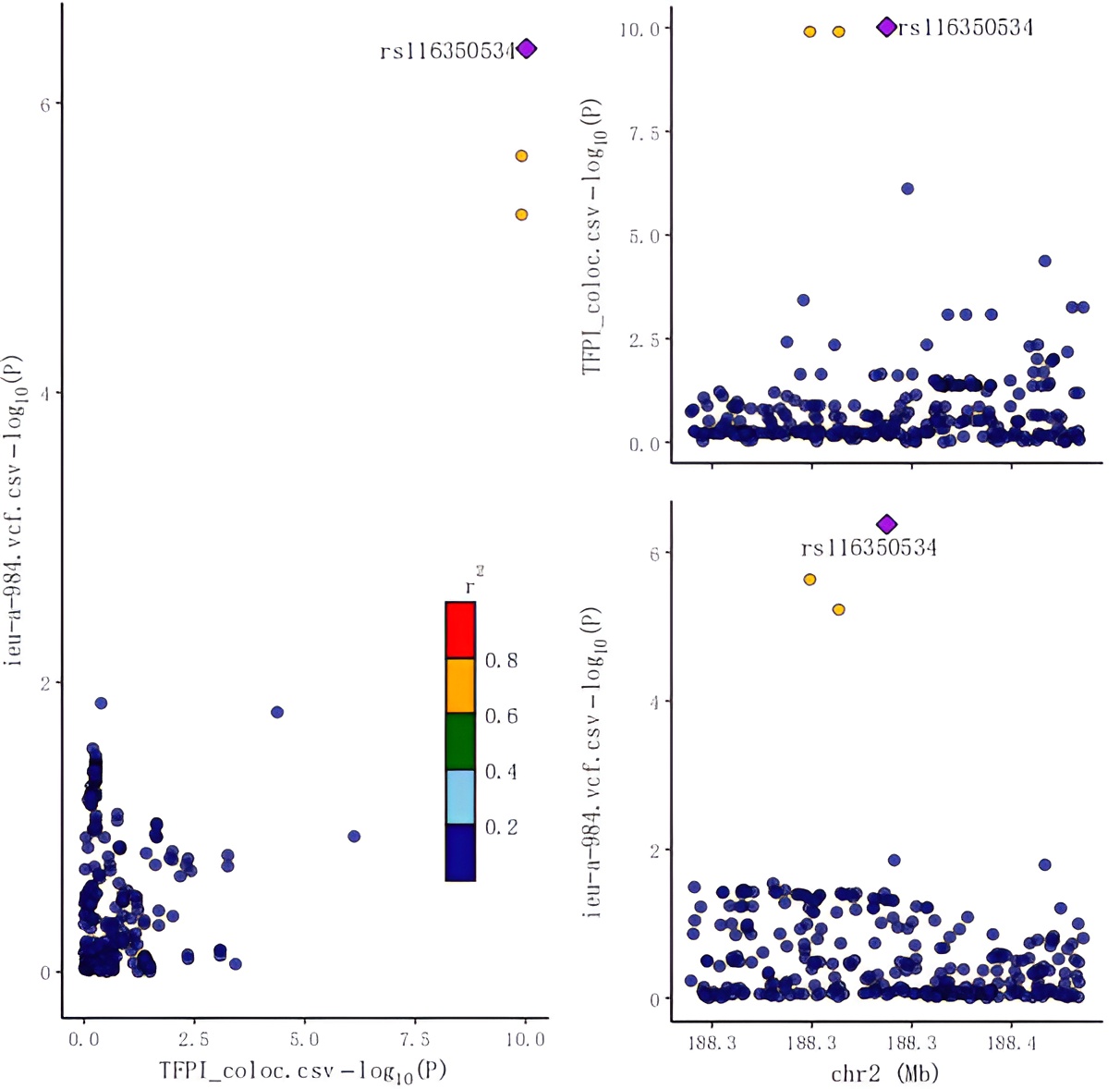


(i) VWC2


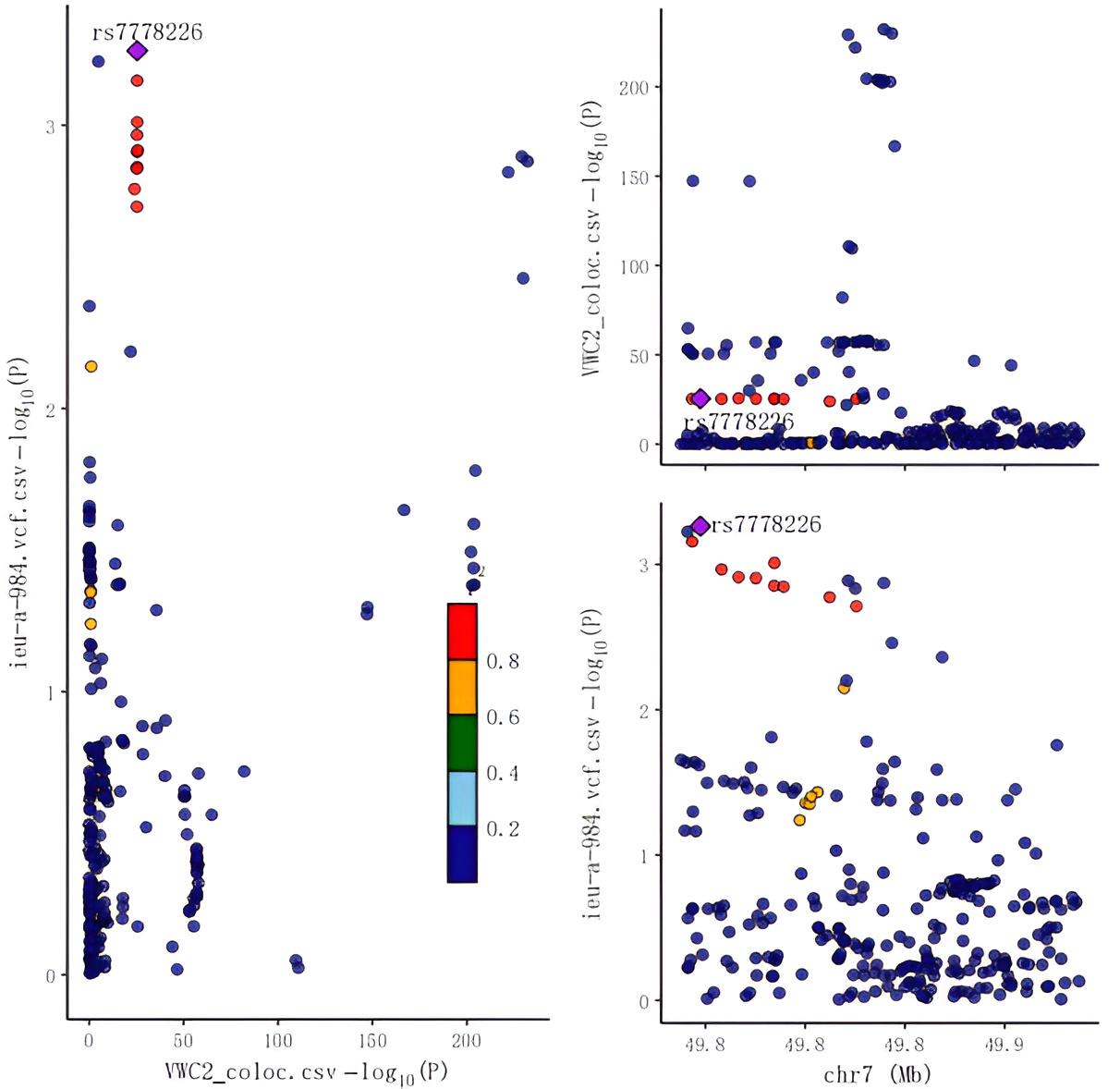


**Figure S5 Colocalization plots of pQTLs and genetic associations of LUAD.** Colocalization analysis of plasma proteins for ALAD (a), FLT1 (b) , ICAM5 (c), MDGA2 (d), NTM (e), PMM2 (f), PTGFRN (g), TFPI (i), and VWC2 (j), respectively. Diamond purple points represented the SNP that with the minimal sum of *P* value in corresponded protein GWAS and LUAD GWAS. pQTL: protein quantitative trait loci; LUAD: lung adenocarcinoma; SNP: single nucleotide polymorphism; GWAS: genome-wide association studies.


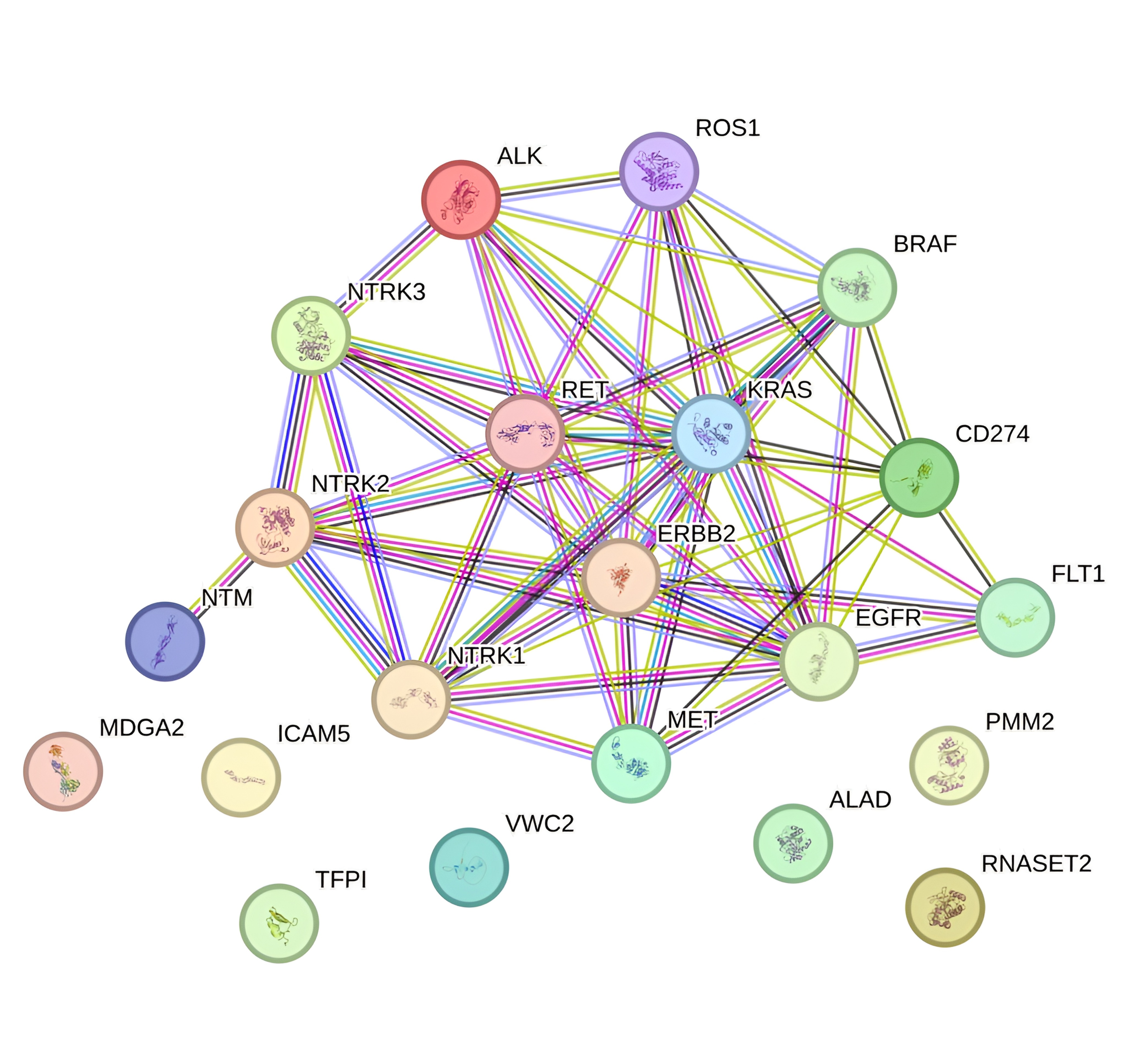


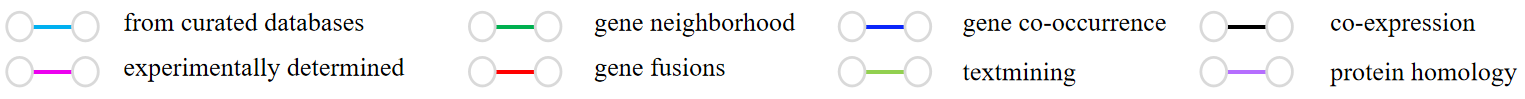


**Figure S6** Protein-protein interaction network among the causal proteins and current lung adenocarcinoma medications targets.
